# The coronavirus macrodomain is required to prevent PARP-mediated inhibition of virus replication and enhancement of IFN expression

**DOI:** 10.1371/journal.ppat.1007756

**Published:** 2019-05-16

**Authors:** Matthew E. Grunewald, Yating Chen, Chad Kuny, Takashi Maejima, Robert Lease, Dana Ferraris, Masanori Aikawa, Christopher S. Sullivan, Stanley Perlman, Anthony R. Fehr

**Affiliations:** 1 Department of Microbiology and Immunology, University of Iowa, Iowa City, IA, United States of America; 2 Department of Molecular Biosciences, University of Texas, Austin, TX, United States of America; 3 Center for Interdisciplinary Cardiovascular Sciences, Cardiovascular Division, Brigham and Women’s Hospital, Harvard Medical School, Boston, MA, United States of America; 4 McDaniel College, Westminster, MD, United States of America; 5 Department of Molecular Biosciences, University of Kansas, Lawrence, KS, United States of America; Institute for Virology, GERMANY

## Abstract

ADP-ribosylation is a ubiquitous post-translational addition of either monomers or polymers of ADP-ribose to target proteins by ADP-ribosyltransferases, usually by interferon-inducible diphtheria toxin-like enzymes known as PARPs. While several PARPs have known antiviral activities, these activities are mostly independent of ADP-ribosylation. Consequently, less is known about the antiviral effects of ADP-ribosylation. Several viral families, including Coronaviridae, Togaviridae, and Hepeviridae, encode for macrodomain proteins that bind to and hydrolyze ADP-ribose from proteins and are critical for optimal replication and virulence. These results suggest that macrodomains counter cellular ADP-ribosylation, but whether PARPs or, alternatively, other ADP-ribosyltransferases cause this modification is not clear. Here we show that pan-PARP inhibition enhanced replication and inhibited interferon production in primary macrophages infected with macrodomain-mutant but not wild-type coronavirus. Specifically, knockdown of two abundantly expressed PARPs, PARP12 and PARP14, led to increased replication of mutant but did not significantly affect wild-type virus. PARP14 was also important for the induction of interferon in mouse and human cells, indicating a critical role for this PARP in the regulation of innate immunity. In summary, these data demonstrate that the macrodomain is required to prevent PARP-mediated inhibition of coronavirus replication and enhancement of interferon production.

## Author summary

ADP-ribosylation, an understudied post-translational modification, facilitates the host response to virus infection. Several viruses, including all members of the coronavirus family, encode a macrodomain to reverse ADP-ribosylation and combat this immune response. As such, viruses with mutations in the macrodomain are highly attenuated and cause minimal disease *in vivo*. Here, using primary macrophages and mice infected with a pathogenic murine coronavirus, we identify PARPs, specifically PARP12 and PARP14, as host cell ADP-ribosylating enzymes important for the attenuation of these mutant viruses and confirm their importance using inhibitors and siRNAs. These data demonstrate a broad strategy of virus-host interactions and indicate that the macrodomain may be a useful target for antiviral therapy.

## Introduction

ADP-ribosylation is the post-translational covalent addition of a single (mono-ADP-ribosylation or MARylation) or multiple (poly-ADP-ribosylation or PARylation) subunits of ADP-ribose from NAD^+^ to a protein. This process is catalyzed by intracellular poly(ADP-ribose) polymerases (PARPs) also known as diphtheria toxin-like ADP-ribosyltransferases (ARTDs), although extracellular cholera toxin-like ADP-ribosyltransferases (ARTCs) and some sirtuins also catalyze ADP-ribosylation [1]. Humans encode 17 PARPs, while mice encode 16. Four (PARP1, PARP2, PARP5a, and PARP5b) are PARylating, while the rest are MARylating or nonenzymatic [2]. Like many post-translational modifications, ADP-ribosylation is reversible by enzymes such as poly(ADP-ribose) glycohydrolase (PARG), ADP-ribosylhydrolases (ARHs), and macrodomains [3–7].

ADP-ribosylation alters the structure and function of the substrate protein and has been implicated in several processes including DNA damage repair, cellular stress response, and virus infection [8]. For instance, PARylating PARPs, such as PARP1/2 and PARP5a/b regulate several nuclear processes such as DNA repair, transcription, and Wnt pathway activation [9, 10]. Mono-ADP-ribosylating PARPs also play a variety of roles in cell biology. For example, PARP16 is required for activation of ER-stress pathways [11], PARP14 binds to STAT-6 and enhances IL-4-dependent gene expression [12–15], PARP9 augments IFNγ-dependent gene expression in macrophages [15], and an unknown ADP-ribosylating enzyme inhibits RNAi following stress responses or poly(I:C) treatment [16–18]. In addition, two sirtuins, SIRT4 and SIRT6, use ADP-ribosylation to inhibit glutamate dehydrogenase and promote DNA repair respectively [19, 20].

PARPs have evolved rapidly, which may reflect their involvement in virus infections [21, 22]. Consistent with this, several PARPs are known ISGs (interferon stimulated genes), and many PARPs have been shown to be antiviral. PARP13, also called zinc antiviral protein (ZAP), inhibits replication of multiple classes of viruses, including retroviruses [23, 24], alphaviruses [22, 25, 26], and filoviruses [27] by binding to viral RNA and recruiting the RNA-degrading exosome complex [28]. ZAP was also found to be required for the ADP-ribosylation and subsequent degradation of influenza A virus proteins PA and PB2 despite being catalytically inactive [29]. Atasheva et al. demonstrated that exogenous expression of PARPs 7, 10, and 12 had inhibitory effects on protein translation and on Venezuelan equine encephalitis virus (VEEV) virus replication using ADP-ribosylation-dependent and -independent mechanisms [30]. This group and others demonstrated that PARP12 could also inhibit vesicular stomatitis virus (VSV), Rift Valley fever virus (RVFV), and encephalomyocarditis virus (EMCV) [31, 32]. PARP12 has been further shown to restrict Zika virus replication by promoting the degradation of viral proteins in an ADP-ribosylation-dependent manner [33]. PARP9 has been shown to complex with the DTX3L ubiquitin ligase to ubiquitinate the host histone H2BJ to enhance IFN signaling, resulting in the inhibition of RNA virus replication [34]. This complex also targets the EMCV 3C protease for ubiquitination and degradation [34]. While PARP9 was important for these activities, whether its ADP-ribosylating activity is required is unclear. In other cases, ADP-ribosylation is important for efficient virus replication as PARP1 inhibitors restrict the replication of several viruses such as herpesviruses, adenoviruses, and HIV [35–37]. PARP7 has both anti- and pro-viral activities as it binds to and induces degradation of Sindbis virus RNA [30, 38] but also promotes influenza A virus infection by ADP-ribosylating TBK1, which inhibits type I IFN (IFN-I) production [39]. Finally, sirtuins 1–7, including the ADP-ribosylating sirtuins SIRT4 and SIRT6, were shown to inhibit the replication of a wide variety of DNA and RNA viruses in MRC-5 cells [18]. However, the mechanism of viral inhibition by sirtuins and whether ADP-ribosylation is involved remains unknown.

All Togaviridae, Coronaviridae, and Hepeviridae encode for a macrodomain protein that can remove ADP-ribose from proteins *in vitro* [40–42]. Several residues have been identified to be important for macrodomain activity, most of which fall in the ADP-ribose binding pocket [43]. Recombinant alphaviruses and hepatitis E virus (HEV) with mutations in these residues generally do not replicate well, while macrodomain-mutant coronaviruses generally replicate normally in tissue culture cells but are highly attenuated *in vivo* [40, 41, 44–50]. Collectively, these results suggest that viral macrodomains counter cellular ADP-ribosylation, but whether PARPs, ARTCs, sirtuins, or other unknown ADP-ribosyltransferases mediate ADP-ribosylation leading to the attenuation of macrodomain-mutant viruses is still unknown.

Coronaviruses (CoVs) are enveloped positive-sense RNA viruses that cause severe disease in several mammalian species. Some, such as porcine epidemic diarrhea virus and porcine delta coronavirus, cause severe disease in agriculturally important animals, while others, such as severe acute respiratory syndrome (SARS)-CoV and Middle East respiratory syndrome (MERS)-CoV, cause lethal human diseases [51]. Mouse hepatitis virus strain JHMV (termed MHV herein) causes acute and chronic demyelinating encephalomyelitis and is the prototypical CoV used in many studies [52]. CoVs maintain several proteins that are important for blocking the innate immune response, including enzymes such as an O-methyltransferase (nsp-(nonstructural protein)16), a deubiquitinase (DUB) (nsp3), an endoribonuclease (nsp15), and an ADP-ribosylhydrolase, the aforementioned macrodomain (nsp3) [53–57]. Accordingly, the SARS-CoV macrodomain-mutant virus was shown to induce a robust pro-inflammatory cytokine response following infection both *in vitro* and *in vivo* [40]. In addition, SARS-CoV and human CoV 229E macrodomain-mutant viruses had increased sensitivity to IFN-I treatment in cell culture, demonstrating that the CoV macrodomain counters antiviral activities of ISGs [48]. Together, these studies suggest that IFN-stimulated ADP-ribosylation is countered by the conserved CoV macrodomain. Here, we show that PARP inhibitors specifically enhance the replication of MHV and decrease IFN production during macrodomain-mutant virus infection, further implicating the macrodomain in countering IFN-induced PARP-mediated antiviral ADP-ribosylation.

## Results

### The enzymatic activity of the MHV macrodomain is required for efficient replication in CD11b+ cells *in vivo*

Mice infected with neurovirulent MHV develop lethal encephalitis [52]. To study the role of the viral macrodomain in MHV-induced neurological disease, we previously created a recombinant virus containing an alanine mutation of a highly conserved asparagine residue (N1347A; herein denotated as N1347A MHV or virus). The location of the macrodomain within nsp3 of MHV and the specific location of this mutation have been previously reported [40, 58]. This asparagine residue is present in all enzymatically active macrodomains, and the asparagine-to-alanine mutation either reduces (CHIKV, HEV) or abolishes (SARS-CoV) the ADP-ribosyl hydrolase activity of viral macrodomains [40–42]. Structurally, the location of this residue within the protein is highly conserved among CoV macrodomains and appears to coordinate the 2’ OH of the distal ribose to influence ADP-ribose binding, catalysis, or both [59, 60].

N1347A MHV replicates poorly and does not cause disease in mice, indicating the importance of this residue for macrodomain function [50]. Macrophages play a central role in this protection as infection with N1347A virus caused severe disease if microglia were depleted from the brain [61]. To directly assess replication of the mutant virus in macrophages *in vivo*, we purified CD11b+ cells (80–90% purity, S1A Fig) from the brains of mice infected with wild-type (WT) or N1347A MHV containing eGFP in place of ORF4 [50]. Of note, ORF4 is not required for optimal virus replication *in vitro* or *in vivo* [62]. Herein, WT virus refers to the previously described *rev*N1347 virus where the WT macrodomain sequence was reinserted into the N1347A MHV BAC clone [50]. Similar to results found in whole brain [50], N1347A virus replication, measured by viral genomic RNA (gRNA) content, was reduced compared to that of WT virus in isolated brain CD11b+ cells (S1B Fig). Because the macrodomain is predicted to counter PARP-mediated ADP-ribosylation, we also analyzed whether PARP expression changed after infection. Consistent with a role for ADP-ribosylation, several PARPs were highly upregulated in these cells following infection with either WT or N1347A virus (S1C Fig).

### N1347A MHV replicates poorly and induces an increased IFN response in bone marrow-derived macrophages

**Fig 1:**
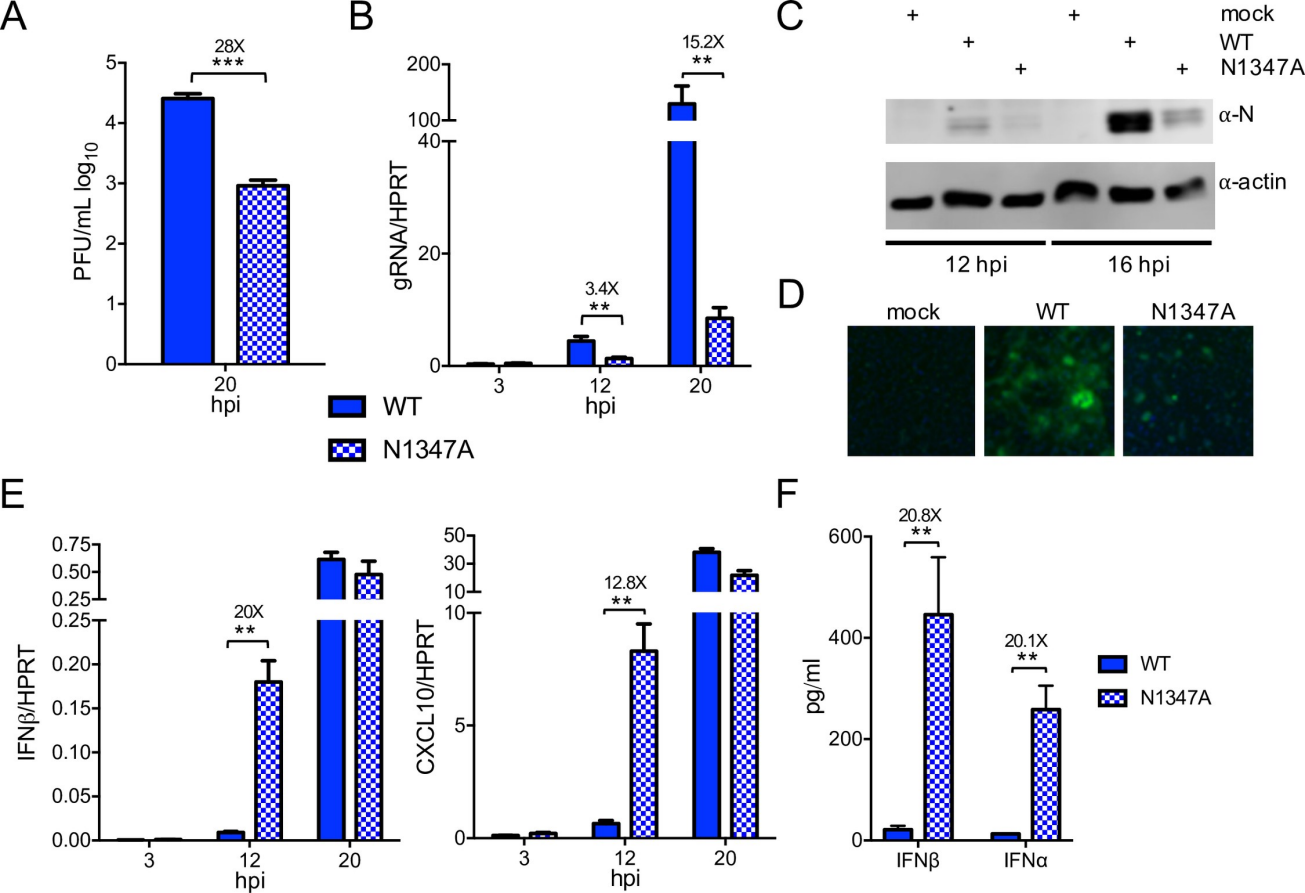
N1347A MHV replicated poorly and induced an increased IFN response in BMDMs. (A-D) BMDMs were infected with WT or N1347A MHV and analyzed for virus replication. (A,B) Virus titers (A) and genomic RNA (gRNA) levels (B) were determined by plaque assay and RT-qPCR with primers specific for nsp12 (normalized to HPRT) respectively. (C,D) BMDMs were infected as described above, and at 14 hpi viral protein levels were determined by immunoblotting infected cell lysates with anti-nucleocapsid protein (N) antibody (C) or visualized by fluorescence microscopy (D). (E) Infected BMDMs were collected at indicated time points, and RNA levels were determined by RT-qPCR with primers specific for each transcript and normalized to HPRT. (F) BMDMs were infected as described above, and at 12 hpi, supernatants were collected and analyzed for IFN⍺ and IFNβ protein levels by ELISA. The data in (A-E) are obtained from one experiment representative of two independent experiments; n = 3 biological replicates. The data in (F) are combined from two independent experiments; WT, n = 6; N1347A, n = 7. Numbers above bars represent fold difference between WT and N1347A.

To date, no cell culture system exists in which a CoV macrodomain-mutant virus has a robust growth defect. Since brain-derived CD11b+ cells are not practical for molecular studies, we next examined whether bone marrow-derived macrophages (BMDMs) could recapitulate the replication deficiency of N1347A MHV seen *in vivo*. To this end, we harvested murine bone marrow cells, differentiated them into macrophages, and infected these BMDMs with WT and N1347A virus at a low multiplicity of infection (MOI) (Fig 1). At 20 hours post infection (hpi), BMDMs infected with N1347A virus had >10-fold lower titers and gRNA levels than those infected with WT virus (Fig 1A and 1B). Furthermore, total viral protein levels were noticeably decreased in N1347A virus-infected cells when measured by immunoblotting for nucleocapsid (N) protein (Fig 1C) or by visually analyzing virus-encoded GFP expression and syncytia formation by fluorescence microscopy (Fig 1D). Unfortunately, the large syncytia formed by infected cells made quantitative flow cytometric analysis of GFP-expressing cells unfeasible.

Previously, we observed a diminished innate immune response in the brains of mice infected with N1347A virus [50], likely reflecting diminished virus replication. To determine if inactivation of the macrodomain in MHV also inhibits the innate immune response in infected BMDMs, we quantified IFN-I and cytokine production after infection with either WT or N1347A virus. However, in contrast to the results seen in infected mice, both CXCL-10 and IFNβ transcript levels and secreted levels of IFNα and IFNβ protein were significantly increased at 12 hpi in BMDMs infected with N1347A virus compared to levels in WT virus-infected samples (Fig 1E and 1F), suggesting that the CoV macrodomain inhibits the innate immune response in infected BMDMs.

### Diminished replication of N1347A MHV does not require enhanced IFN-I production but is dependent on IFN-I signaling

**Fig 2:**
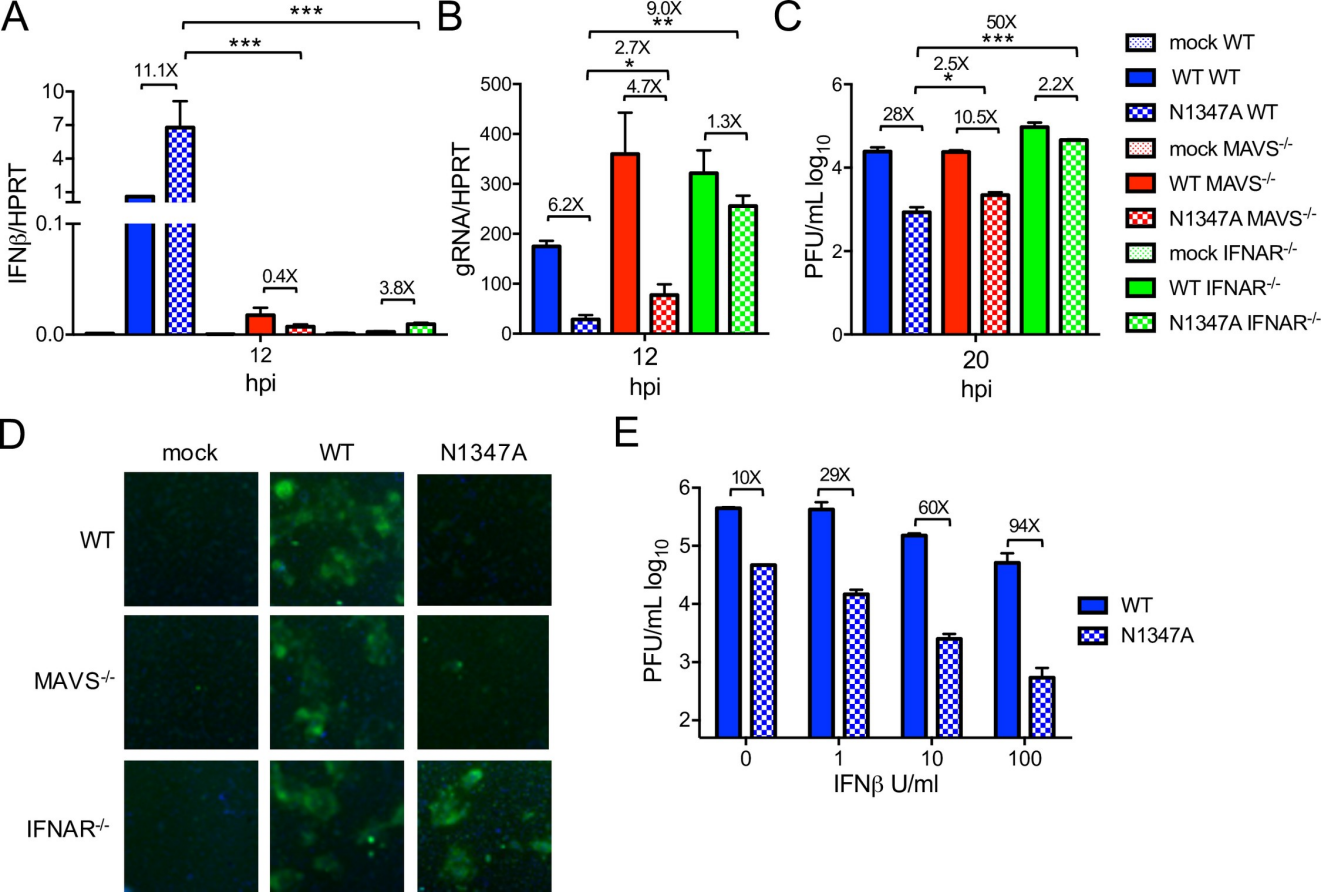
Attenuation of N1347A MHV replication requires IFN-I signaling. (A-D) BMDMs from WT, MAVS^-/-^, or IFNAR^-/-^ mice were mock infected or infected with WT or N1347A MHV, and cells were collected at indicated timepoints. RNA levels were determined by RT-qPCR with primers specific to IFNβ (A) or genomic RNA (B), viral titers were determined by plaque assay (C), and viral protein expression was assessed by fluorescence microscopy (D). The data in (A-D) show one experiment representative of at least two independent experiments; n = 3. (E) BMDMs were pretreated with indicated amounts of IFNβ for 8 hours, media was removed, and the cells were infected with WT or N1347A virus. Cells were collected at 18 hpi, and virus titers were determined by plaque assay. The data in (E) show one experiment representative of three experiments; n = 3. Numbers above bars represent fold difference between WT and N1347A or between WT, MAVS^-/-^, and IFNAR^-/-^ cells infected with N1347A virus.

To determine if restriction of N1347A MHV replication is due to mechanisms upstream or downstream of IFN-I signaling, we infected BMDMs isolated from WT, MAVS^-/-^ (mitochondrial antiviral signaling protein), and IFNAR^-/-^ (interferon α/β receptor) mice with WT and N1347A MHV. Loss of MAVS or IFNAR greatly reduced IFNβ mRNA levels compared to those in WT cells (Fig 2A). Furthermore, the N1347A MHV-mediated increase in IFNβ mRNA seen in WT cells was ablated in MAVS^-/-^ cells (Fig 2A). However, while the replication deficiency of N1347A virus in WT BMDMs was retained in MAVS^-/-^ cells, it was largely rescued in IFNAR^-/-^ cells as measured by genomic RNA levels, viral titers, and visualized by GFP expression (Fig 2B–2D). To determine if these *in vitro* findings correlate with virulence, we infected WT, MAVS^-/-^, and IFNAR^-/-^ mice intranasally with WT and N1347A virus (S2 Fig). N1347 virus-infected WT and MAVS^-/-^ mice exhibited 100% survival and minimal differences in weight loss. In contrast, 60% of IFNAR^-/-^ mice infected with N1347A virus succumbed to the infection and exhibited weight loss similar to that induced by WT virus. To further confirm that the factor(s) limiting N1347A MHV replication is downstream of IFN-I, we pretreated WT BMDMs with different doses of IFNβ for 8 hours and then infected cells with WT or N1347A virus (Fig 2E). Increasing amounts of IFNβ further reduced N1347A virus titers compared to that of WT, demonstrating that ISGs restrict N1347A MHV replication.

### Several PARPs are highly expressed during infection and in response to IFN stimulation in BMDMs

**Fig 3:**
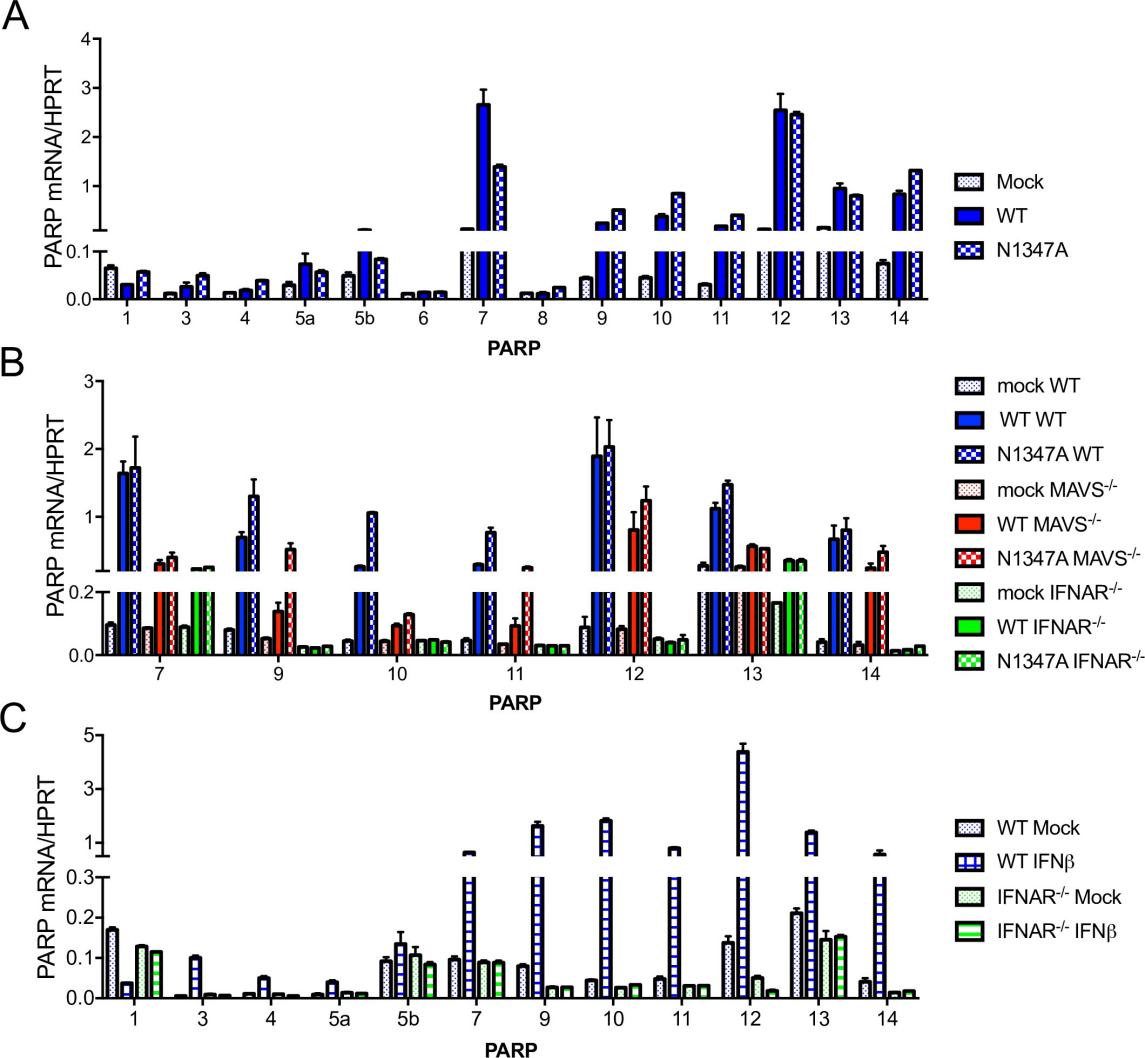
Several PARPs are highly upregulated by MHV infection and IFN treatment in BMDMs. (A) BMDMs were infected with WT or N1347A MHV and collected at 18 hpi. PARP expression was determined by RT-qPCR using primers listed in S3 Table and normalized to HPRT. PARP2 and PARP16 were undetectable and are not shown. (B) BMDMs isolated from WT, MAVS^-/-^, or IFNAR^-/-^ mice were infected with WT or N1347A virus. Cells were collected at 18 hpi, and RNA levels of selected PARPs were determined by RT-qPCR and normalized to HPRT mRNA levels. (C) BMDMs from WT or IFNAR^-/-^ mice were mock treated or treated with 1000 U IFNβ for 8 h, and RNA levels were determined by RT-qPCR and normalized to HPRT. PARPs 6 and 8 fell below the limit of detection and are not shown. The data in (A-C) show one experiment representative of two independent experiments; n = 3.

The macrodomain is an ADP-ribosylhydrolase, raising the possibility that PARP enzymes are responsible for the attenuation of the N1347A virus. PARPs are known ISGs, and the lack of upregulation of these proteins in IFNAR^-/-^ cells or mice could explain the restoration of N1347A MHV-specific phenotypes. First, we determined whether PARPs were upregulated during infection by measuring PARP mRNA levels in BMDMs infected with WT and N1347A virus (Fig 3A). Several PARP family members, including PARPs 7 and 9–14, were upregulated in both WT and N1347A virus-infected BMDMs compared to mock-infected cells. Of note, our PARP13 primers were designed to detect all isoforms of PARP13, and we were unable to detect PARP2 or PARP6. Furthermore, the expression of these upregulated PARPs were also increased in infected MAVS^-/-^ cells, while the expression of PARPs 9–12 and 14 were not increased in infected IFNAR^-/-^ cells (Fig 3B), suggesting these PARPs are ISGs, consistent with previous reports [31, 32, 63, 64]. We confirmed this by treating WT and IFNAR^-/-^ BMDMs with IFNβ and measuring PARP mRNA levels (Fig 3C). As expected, PARPs 9–12 and 14, in addition to PARPs 3, 4, and 5a, were upregulated following IFNβ stimulation in WT but not in IFNAR^-/-^ BMDMs, consistent with previous studies [31, 65]. Interestingly, PARP7 and PARP13 were induced by IFNβ but were also induced in infected IFNAR^-/-^ cells, demonstrating that, while these PARPs are ISGs, they are also regulated by additional mechanisms during infection (Fig 3B and 3C). We conclude that most PARPs are ISGs in primary murine macrophages and that PARPs 7 and 9–14 are highly expressed following CoV infection.

### PARP activity restricts N1347A MHV replication and enhances the innate immune response

**Fig 4:**
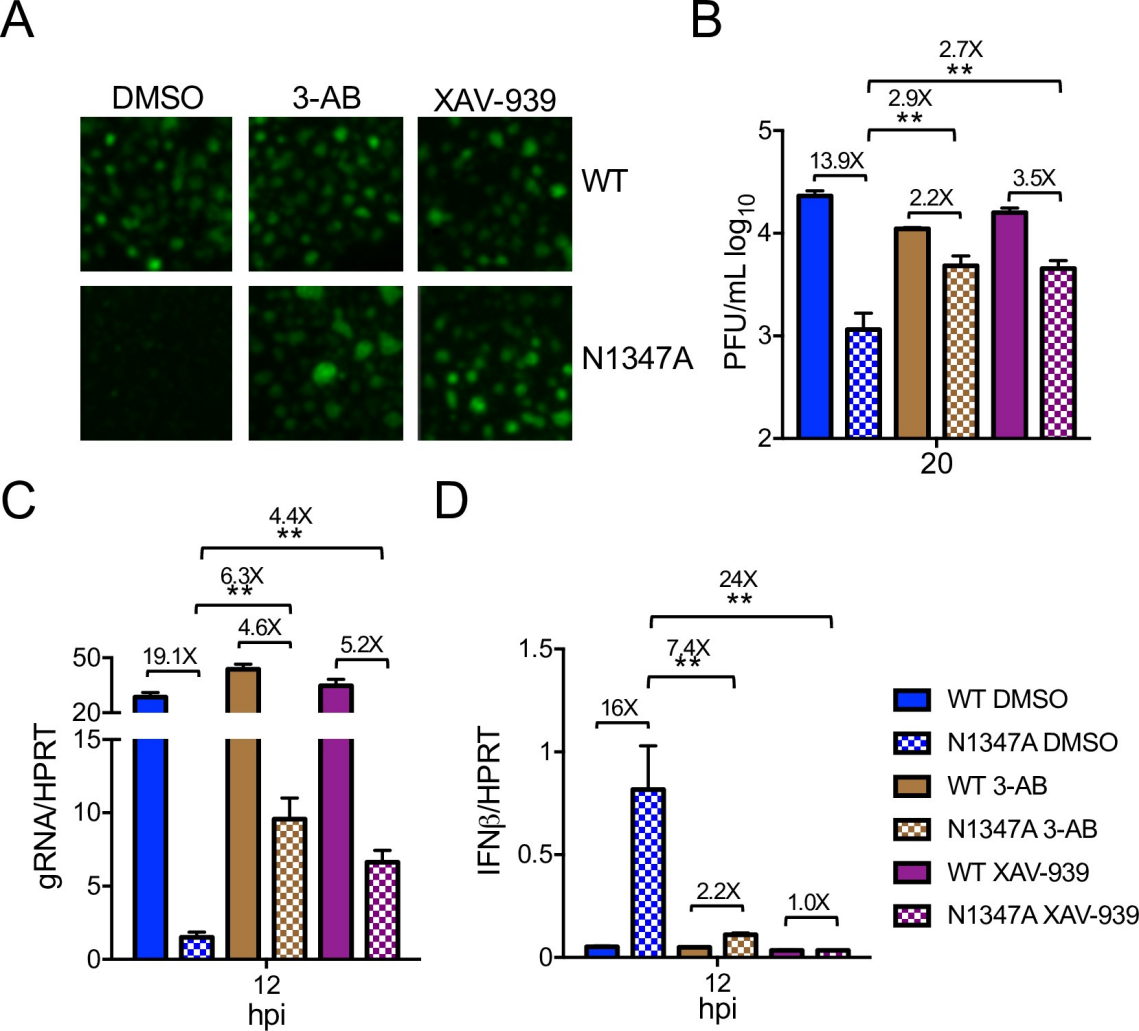
PARP catalytic activity is required for inhibition of replication and enhanced IFN production during N1347A MHV infection. (A-D) BMDMs were infected with WT or N1347A MHV and treated with vehicle (0.25% DMSO), 5 mM 3-AB, or 10 μM XAV-939 following a 1-hour adsorption phase. Cells were either fixed at 16 hpi and analyzed for virus-encoded GFP expression by fluorescence microscopy (A); collected at 20 hpi, and viral titers determined by plaque assay (B); or collected at 12 hpi, and RNA levels determined by RT-qPCR with primers specific to genomic RNA (C) or IFNβ (D). The data in (A-D) show one experiment representative of two independent experiments; DMSO, n = 6; 3-AB and XAV-939, n = 3. Numbers above bars represent fold difference between WT and N1347A or between DMSO- and inhibitor-treated cells infected with N1347A virus.

To directly test whether PARPs inhibit CoV replication and facilitate IFN-I production in the absence of macrodomain ADP-ribosylhydrolase activity, we infected cells with WT and N1347A MHV prior to treatment with PARP inhibitors 3-aminobenzamide (3-AB) and XAV-939 (Fig 4). 3-AB is a general PARP inhibitor, while XAV-939 was developed as a PARP5a/b inhibitor, but at higher concentrations it inhibits most, if not all, PARPs [66]. These inhibitors did not affect cell growth or metabolism at the concentration used in this study but diminished cellular PARylation, demonstrating efficacy (S3A & S3C Fig). Importantly, both inhibitors significantly increased N1347A virus replication compared to vehicle treatment as visualized by GFP expression or measured by viral titers or genomic RNA levels (Fig 4A–4C). Further, levels of IFNβ transcript produced in N1347A virus-infected cells treated with inhibitors were reduced to levels seen in WT virus-infected cells (Fig 4D). Importantly, neither inhibitor had a significant effect on replication or IFN production in WT virus-infected cells, suggesting that PARPs are potentially counteracted by macrodomain activity during WT infection. As these inhibitors are known to target the PARP catalytic site [66], these data indicate that PARP-catalyzed ADP-ribosylation is responsible for decreased replication and increased IFNβ production during N1347A MHV infection.

### PARP12 and PARP14 are required for the restriction of mutant virus replication

**Fig 5:**
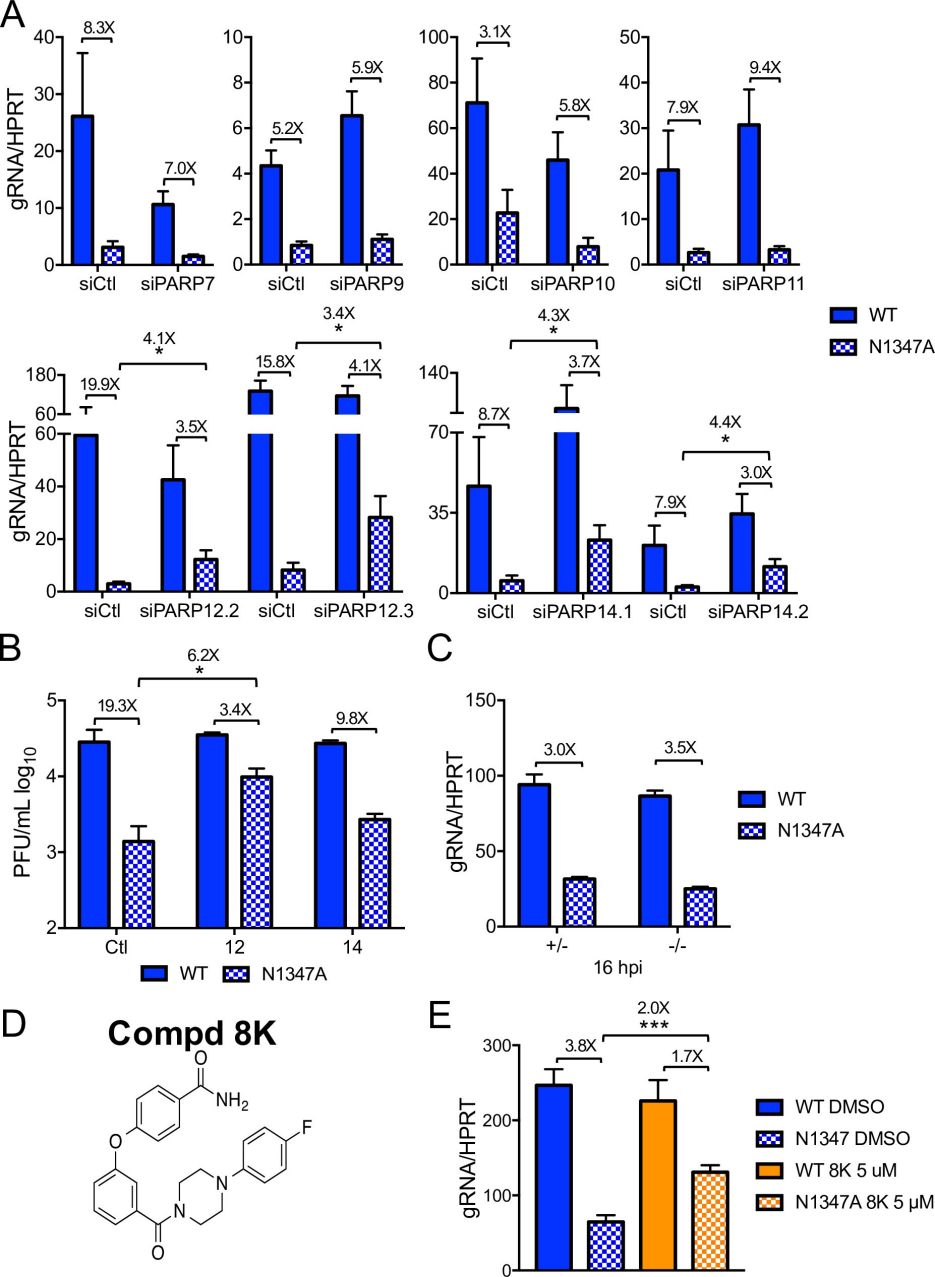
PARP12 and PARP14 restrict N1347A MHV replication in BMDMs. (A,B) BMDMs were transfected with control siRNA (siCtl) or PARP-specific siRNA as described in Methods. Approximately 28 hours later, cells were infected with WT or N1347A MHV and collected at 18–22 hpi. RNA levels were determined by RT-qPCR (A), and viral titers were determined by plaque assay (B). The data in (A) represent the combined results of two to five experiments; n = 9 for siPARP7, n = 6 for siPARP9, n = 6 for siPARP10, n = 12 for siPARP11, n = 15 for siPARP12.2, n = 9 for siPARP12.3, n = 12 for siPARP14.1, n = 12 for siPARP14.2. The results in (B) show one experiment representative of two independent experiments. (C) PARP14^-/-^ or PARP14^+/-^ littermate control BMDMs were infected with WT or N1347A virus, cells were collected at 15 hpi, and gRNA levels were determined by RT-qPCR. The data in (C) show one experiment representative of four independent experiments. (D) Structure of PARP14 inhibitor compound 8K. (E) BMDMs were infected with WT or N1347A virus and treated with 5 μM compound 8K or vehicle (DMSO) following a 1-hour adsorption phase. Cells were collected at 18 hpi, and gRNA levels were determined by RT-qPCR. The data in (E) represent the combined results of four experiments; n = 12. Numbers above bars represent fold difference between WT and N1347A or between siPARP/siCtl or inhibitor/DMSO-treated cells infected with N1347A virus.

To determine which individual PARP(s) restricts replication of N1347A MHV, we transfected BMDMs with siRNAs for the most highly expressed PARPs and examined the effects on WT and N1347A virus replication. We were unable to reliably knockdown PARP13 expression, so this PARP was excluded from our analysis. Knockdown of all other tested PARP mRNAs in both WT and N1347A MHV-infected BMDMs was observed (S4 Fig). Knockdown of PARPs 7, 9, 10, and 11 did not significantly increase WT or N1347A virus gRNA levels over that of control siRNA-transfected N1347A virus-infected cells (Fig 5A, top row). In contrast, two independent siRNAs directed toward PARP12 and PARP14 significantly rescued N1347A virus gRNA levels without having a significant effect on WT virus (Fig 5A, bottom row). Viral titers were also increased in cells transfected with siPARP12.2 or with siPARP14.1, although the increased replication of N1347A virus in PARP14 knockdown cells did not reach statistical significance (Fig 5B). To further examine the role of PARP14 in N1347A MHV infection, we infected BMDMs harvested from PARP14^-/-^ and PARP14^+/-^ mice (S5 Fig). N1347A virus replication was not significantly different in PARP14^-/-^ and PARP14^+/-^ cells (Fig 5C), suggesting that other PARPs or factors important for restricting replication may have compensated for or were lacking in the congenital absence of PARP14. In an effort to resolve these differences, we utilized a recently developed PARP14 inhibitor, compound 8K, which targets the MARylating catalytic site of PARP14 (Fig 5D) [67]. While compound 8K did not affect cell viability or metabolism or inhibit global cellular PARylation (S3B & S3C Fig), it significantly restored replication of N1347A virus in BMDMs (Fig 5E). In general, these results support a role for PARP12 and PARP14 in blocking N1347A MHV replication. However, whether or not the catalytic domain of PARP12 is required for this role will require further validation.

### PARP14 is required for maximal IFN production in response to CoV infection or poly(I:C) treatment

**Fig 6:**
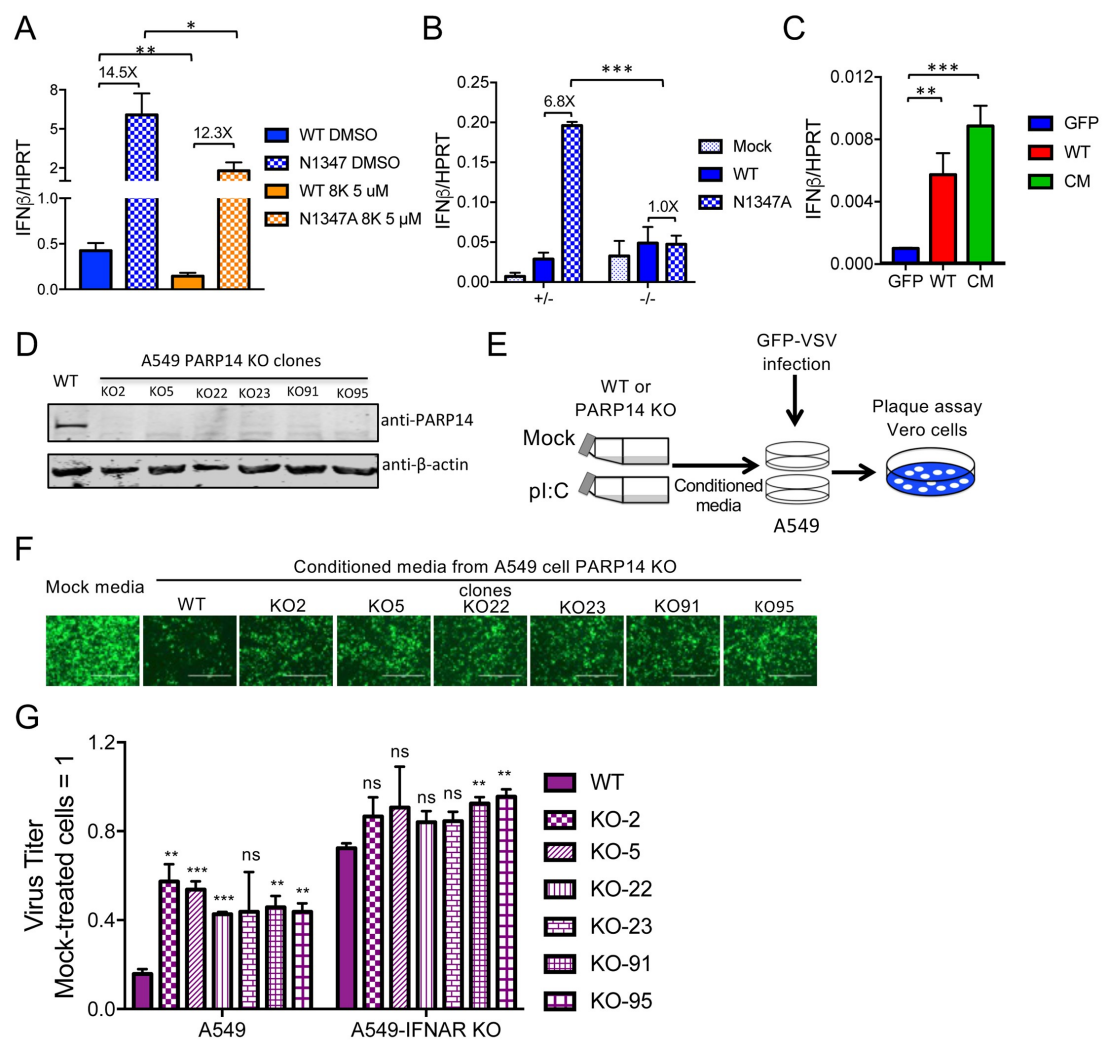
PARP14 is required for an efficient IFN response following infection or poly(I:C) induction. (A) BMDMs were infected with WT or N1347A MHV and treated with 5 μM compound 8K or vehicle (DMSO) following a 1-hour adsorption phase. Cells were collected at 12 hpi, and IFNβ mRNA levels were determined by RT-qPCR. The data in (A) represent the combined results of three experiments; n = 9. (B) PARP14^-/-^ or PARP14^+/-^ littermate control BMDMs were infected with WT or N1347A virus. Cells were collected at 12 hpi, and IFNβ mRNA levels were determined by RT-qPCR. The data in (B) show one experiment representative of four independent experiments. (C) DBT cells were transfected with plasmid expressing GFP, PARP14, or a PARP14 catalytic mutant (CM). Cells were collected at 24 hours after transfection, and IFNβ mRNA levels were determined by RT-qPCR. The data in (C) show one experiment representative of two independent experiments; n = 3. (D) Six clones of CRISPR/Cas9-gRNA-mediated PARP14 KO A549 cells or gRNA-NC-transduced (irrelevant gRNA) control A549 cells were collected and analyzed by immunoblotting for PARP14 protein. (E) Schematic diagram illustrating the antiviral conditioned media assay (see Methods for details). (F,G) At 16 hpi, VSV replication on WT or IFNAR KO A549 cells was analyzed by fluorescence microscopy (F), and titers were determined by plaque assay on Vero cells (G). The images in (F) are of a single experiment representative of three independent experiments. Titers in (G) were normalized to those in cells receiving mock-treated conditioned media. The data in (G) show the combined results of three independent experiments; n = 3. x-axis labels represent VSV-infected cells. p-values represent comparisons of each poly(I:C) (pI:C)-treated PARP14 KO cell conditioned media to poly(I:C)-treated WT cell conditioned media used to pretreat WT or IFNAR KO A549 cells. Numbers above bars represent fold difference between WT and N1347A or between siPARP/siCtl or inhibitor/DMSO-treated cells infected with N1347A virus.

Because PARP14 impacts innate immune signaling pathways [12, 63], we also tested whether the PARP14 inhibitor affects IFN production. We found that, in addition to partially rescuing N1347A MHV replication (Fig 5D), the PARP14 inhibitor 8K caused a reduction in IFNβ mRNA levels in both WT and N1347A virus-infected BMDMs (Fig 6A). Consistent with these results, PARP14^-/-^ in contrast to PARP14^+/-^ cells showed no increase in IFN expression following infection with N1347A virus (Fig 6B). Notably, overexpression of PARP14, but not of GFP, was also sufficient for IFN induction in delayed brain tumor (DBT) cells, which normally express very low, if any, IFN (Figs 6C & S6). Overexpression of a PARP14 mutant with inactivating mutations H1698F, Y1730N, and E1810K in the catalytic triad of the PARP domain (CM, described in [68]) also induced IFN expression in DBT cells following transfection (Fig 6C). This result suggests that PARP14 has both ADP-ribosylation-dependent and -independent mechanisms for regulating the IFN response.

To determine whether PARP14 is also important for IFN induction in human cells, we used an assay based on IFN-mediated inhibition of VSV (vesicular stomatitis virus) replication. We engineered six PARP14 knockout (KO) clones of A549 cells and a pool of PARP14 KO cells in normal human dermal fibroblasts (NHDFs) using lentiCRISPR/CAS9-v2 (Figs 6D & S7A). These cells were transfected with poly(I:C) for 6 hours, at which point the supernatant (conditioned media) was collected and transferred to naïve A549 cells. After a 2-hour incubation, we then infected these cells with an eGFP-expressing mutant recombinant VSV (VSV M51R) that is unable to suppress the innate immune response [69] and measured virus replication by plaque assay on Vero cells to quantify the amount of antiviral cytokines in the conditioned media (Fig 6E). As expected, treatment with conditioned media from poly(I:C)-transfected WT A549 (Fig 6F and 6G) or WT NHDF cells (S7B and S7C Fig) induced a substantial decrease in VSV M51R replication compared to media from mock-transfected cells. In contrast, conditioned media harvested from stimulated PARP14 KO A549 (Fig 6F and 6G) or PARP14 KO NHDF (S7B and S7C Fig) cells showed only partial antiviral activity, resulting in ~3-fold enhanced replication of VSV M51R compared to cells that received conditioned media from stimulated WT cells. To confirm the antiviral activity in the conditioned media is due to IFN-I, we engineered cells functionally knocked out for IFNAR1 and tested whether the conditioned media had any effects on VSV M51R replication in these cells. The addition of exogenous IFNα to IFNAR KO A549 cells had no effect on VSV M51R replication, whereas 10 units of IFNα restricted VSV replication in WT cells, confirming that the IFNAR KO cells were defective for IFN-I signaling (S7D Fig). Conditioned media from both WT and PARP14 KO cells treated with poly(I:C) still had a mild inhibitory effect on VSV M51R replication in IFNAR KO cells, suggesting that antiviral cytokines in addition to IFN-I were present in the media. However, a smaller difference in VSV replication between WT and PARP14 KO conditioned media-treated cells was observed in IFNAR KO recipient cells compared to WT recipient cells (Figs 6G and S7C), indicating that the primary antiviral factor produced by stimulated cells was IFN-I. Taken together, these data indicate that PARP14 is necessary for efficient IFN-I production during CoV infection and poly(I:C) stimulation in both mouse and human cells respectively.

## Discussion

Here we show that PARPs, specifically PARP12 and PARP14, are required to inhibit the replication of a macrodomain-mutant CoV and that PARP14 is also required for optimal IFN expression. Previous reports have shown that CoVs were unable to cause disease in the absence of viral macrodomain ADP-ribosylhydrolase activity and that this attenuation was associated with reduced viral loads and changes in pro-inflammatory cytokine expression *in vivo* [40, 47, 50]. However, it has been difficult to elucidate the details of CoV macrodomain function due to the lack of a cell culture system that recapitulates these phenotypes. Our results showed that BMDMs are useful for this purpose since N1347A MHV replicated poorly and induced a robust IFN response in these cells (Fig 1). BMDMs provide several advantages over other primary cells in that they are easily cultured, can be obtained in large numbers, have a fully functional innate immune response, and are productively infected by MHV [70].

PARPs have both antiviral and immunomodulatory roles [8]. Here we found that pan-PARP inhibitors both decreased IFN production and enhanced the replication of MHV lacking ADP-ribosylhydrolase activity but had no significant effect on WT virus (Fig 4). The antiviral properties of PARPS were dependent upon their ADP-ribosyltransferase activity since these inhibitors target the catalytic site of PARPs. These results suggest that the conserved CoV macrodomain functions to directly counter the activity of cellular PARP enzymes; however, this conclusion will require further validation. In addition, utilizing siRNA knockdown and a PARP14 specific inhibitor, we provide evidence that both PARP12 and PARP14 are necessary to restrict N1347A virus replication (Fig 5A, 5B and 5E). PARP12, which has high sequence similarity to PARP13 (ZAP), has previously been shown to block both cellular and viral protein translation and inhibit virus replication by both ADP-ribosylation dependent and independent mechanisms [30]. On the other hand, PARP14 was not previously shown to inhibit virus replication but rather was shown to modulate both innate and adaptive immune responses [15, 63, 68, 71]. Both proteins are known components of stress granules where they interact with a variety of proteins, including Argonaute 2 and PARP13 (ZAP) [16]. Interestingly, PARP14 was recently shown to ADP-ribosylate PARP13 in human cells [72]. It will be intriguing to determine: i) if PARP12 and PARP14 localize to stress granules during a CoV infection, ii) whether PARP13 is involved in restricting N1347A MHV replication, and iii) how these PARPs specifically impact the CoV lifecycle. Most important will be to identify those proteins that are ADP-ribosylated by PARPs and targeted by the macrodomain during infection to fully understand this important host-virus interaction. Since the macrodomain is a subunit within a large transmembrane viral protein (nsp3), it is likely that the targets for de-ADP-ribosylation are cellular proteins in close vicinity to nsp3. These include proteins located in replication-transcription complexes (RTCs) or other subcellular structures such as P-bodies or stress granules.

Studies with chikungunya virus and Sindbis virus demonstrate that the alphavirus macrodomain also counters cellular ADP-ribosylation as mutants with decreased ADP-ribose binding and enzymatic activity had mild to severe replication defects [44, 45, 73]. However, following infection with chikungunya virus, very little PARP induction was observed in infected cells, and a MARylation inhibitor decreased WT virus replication [44]. Thus, while PARPs play an antiviral role during a CoV infection, their role during alphavirus infection may be more nuanced.

About half of the PARP family members are induced by MHV infection in BMDMs (Fig 3), raising the possibility that PARPs in addition to PARP12 and PARP14 contribute to the antiviral response. Consistent with this, pan-PARP inhibitors reduced N1347A MHV-induced IFN-I levels (Fig 4D) much more effectively than the PARP14-specific inhibitor (Fig 6A). Notably, the expression of PARP7 and PARP13 following infection were upregulated in infected IFNAR^-/-^ BMDMs (Fig 3B), suggesting that they are part of an IFN-independent cellular response to infection. In addition, knockdown of either PARP7 or PARP10 mRNA mildly reduced gRNA production in cells infected with either WT or N1347A virus (Fig 5A), suggesting that they have pro-viral functions in MHV-infected BMDMs, perhaps analogous to alphavirus-infected cells but in contrast to other virus infection models where they are known to be antiviral [30, 38].

Finally, using a recently developed PARP14-specific inhibitor and PARP14^-/-^ BMDMs (Fig 6A and 6B) as well as human PARP14 KO A549 and NHDF cells (Fig 6D–6G, S7A–S7C Fig), we found that PARP14 was required for robust IFN-I production during CoV infection or poly(I:C) stimulation. These data are consistent with a recent report detailing a role for PARP14 in IFN-I induction following LPS stimulation of RAW 264.7 cells and BMDMs [63]. In that study, the absence of PARP14 did not affect IRF-3 translocation to the nucleus but rather altered histone modification and reduced pol II recruitment to specific ISG promoters in the nucleus. Consistent with these data, we also showed that PARP14 overexpression induced IFN-I transcription in DBT cells (Fig 6C). PARP14 with three inactivating mutations in the catalytic triad also induced IFN expression (Fig 6C), suggesting that PARP14 uses both ADP-ribose-dependent and independent mechanisms to regulate the IFN response during CoV infection. This result may not be surprising since a PARP14 fragment without the catalytic domain has been shown to activate STAT-6 dependent transcription, but the catalytic domain was required for maximal activation [12].

Many of the signaling proteins involved in the production of IFN-I and ISGs are known to be regulated by post-translational modifications such as phosphorylation and ubiquitination [74]. Recently, several of these proteins have been demonstrated to be regulated by PARPs as well [13, 15, 39, 63, 75, 76]. These findings, along with our data, unveil previously unknown mechanisms of innate immune regulation and suggest that PARP-dependent ADP-ribosylation will impact several proteins in these pathways. Clearly, the next step will be to identify the targets of individual PARPs and determine precisely how ADP-ribosylation regulates the innate immune response.

## Methods

### Ethics statement

Animal studies were approved by the University of Iowa Institutional Animal Care and Use Committee (IACUC) as directed by the Guide for the Care and Use of Laboratory Animals (Protocol #6071795). Anesthesia or euthanasia were accomplished using isoflurane and ketamine/xylazine or ketamine/xylazine, respectively.

### Cell culture, plasmids, and reagents

Delayed brain tumor (DBT) (propagated in Perlman laboratory since 1983), normal human dermal fibroblasts (NHDF) (ATCC), A549 (ATCC), Vero (a gift provided by Robert Krug, University of Texas at Austin), BHK-21 (a gift provided by Dr. Emin Ulug, University of Texas at Austin) and HeLa cells expressing the MHV receptor carcinoembryonic antigen-related cell adhesion molecule 1 (CEACAM1) (HeLa-MHVR) (a gift from Dr. Thomas Gallagher, Loyola University Chicago) were grown in Dulbecco’s Modified Eagle Medium (DMEM) supplemented with 10% fetal bovine serum (FBS), 100 U/ml penicillin and 100 μg/ml streptomycin. Bone marrow-derived macrophages (BMDMs) sourced from WT, MAVS^-/-^, IFNAR^-/-^, PARP14^+/-^and PARP14^-/-^ C57BL/6 mice were differentiated by incubating cells with 10% L929 cell supernatants and 10% FBS in Roswell Park Memorial Institute (RPMI) media for seven days. Cells were washed and replaced with fresh media every day after the 4^th^ day.

pcDNA3-GFP was previously described [40], pcDNA3-FLAG/PARP14 and pcDNA3-FLAG/PARP14-CM were obtained as a generous gift from Dr. Mark Boothby (Vanderbilt University, Nashville TN), and pSport6-FLAG/PARP12 was obtained as a generous gift from Dr. Oberdan Leo (Universite Libre de Bruxelles, Gosselies, Belgium). Mouse IFNβ and human IFNα was purchased from PBL Assay Science (12401–1 and 11200–1). 3-aminobenzamide (3-AB) (A4161) and XAV-939 (A1877) were purchased from APExBIO. Compound 8K was previously described [67].

### Cell viability

Following differentiation, BMDMs were treated with the indicated compounds for 24 hours. Cell viability was assessed using a Vybrant MTT Cell Proliferation Assay (Thermo Fisher Scientific) following manufacturer’s instructions.

### Mice

Pathogen-free C57BL/6 WT and IFNAR^-/-^ mice were purchased from Jackson Laboratories, and MAVS^-/-^ mice were obtained as a generous gift from Dr. Michael Gale (University of Washington, Seattle, Washington). These mice were bred and maintained in the animal care facility at the University of Iowa. PARP14^-/-^ mice were a generous gift from Dr. Mark Boothby (Vanderbilt University, Nashville, Tennessee) and were bred and maintained in the animal care facility at Harvard Medical School.

### Virus infection

Recombinant WT (rJ^IA^-GFPrevN1347) and N1347A (rJ^IA^-GFP-N1347A) MHV were previously described [50, 77]. Both viruses expressed eGFP. Virus stocks were created by infecting ~1.5×10^7^ 17Cl-1 cells at an MOI of 0.1 plaque-forming units (PFU)/cell and collecting both the cells and supernatant at 20 hpi. The cells were freeze-thawed, and debris was removed prior to collecting virus stocks. Virus stocks were quantified by plaque assay on Hela-MHVR cells. BMDM cells were infected with MHV at an MOI of 0.1 PFU/cell with a 45–60 min adsorption phase. Infected cells were then incubated and collected at the indicated timepoints. Recombinant eGFP-expressing mutant VSV (rM51R-M-EGFP) was obtained from Dr. Douglas Lyles, Wake Forest School of Medicine, Winston-Salem, NC [78]. Virus stocks were amplified in BHK-21 cells and quantified by plaque assay on Vero cells. For mouse infections, 5-8-week-old mice were anesthetized with ketamine/xylazine and inoculated intranasally with 3×10^4^ PFU of virus in 12 μL DMEM. Mice were either monitored for weight loss or were sacrificed at 4–6 days post infection (dpi) to harvest the brain tissue. Brain tissues were homogenized, and leukocytes were isolated as previously described [79].

### CD11b+ cell purification and flow cytometry

CD11b+ leukocytes from brain tissues were purified using CD11b MicroBeads (Miltenyi Biotec) as per manufacturer’s instructions. For surface staining, brain leukocytes were treated with Fc block (CD16/32, 2.4G2) and then incubated with specific mAbs or isotype controls. Monoclonal antibodies used for these studies included CD45-PECy7/FITC (30-F11, Biolegend) and CD11b-e450 (M1/70, Thermo Fisher Scientific). Cells were analyzed using a FACS Verse flow cytometer (BD Biosciences). All flow cytometry data were analyzed using FlowJo software (Tree Star, Inc.).

### Plasmid and siRNA transfection

5×10^5^ DBT cells were transfected with 0.5 μg total plasmid expressing GFP, PARP12, or WT or CM PARP14 using PolyJet *In Vitro* Transfection Reagent (SignaGen Laboratories) as per manufacturer’s protocol. Media was replaced 8 hours after transfection, and cells were incubated for 16 hours before collection.

For siRNA knockdown, DsiRNA oligonucleotides were purchased from Integrated DNA Technologies (IDT). Sequences are listed in S1 Table. Negative control DsiRNA was also purchased from IDT and is listed as a non-specific control. BMDMs were transfected with 50 pmol/ml of siRNA with Viromer BLUE (Lipocalyx) following the manufacturer’s protocol. Media was replaced 4 hours after transfection, and cells were further incubated for 24 hours prior to infection. Three independent siRNAs were acquired for each gene, and the one giving the best knockdown was used for viral replication assays, except for PARPs 12 and 14 assays, which utilized 2 independent siRNAs.

### Generation of PARP14- and IFNAR1-targeted KO cell lines

PARP14 KO A549 and NHDF cells and IFNAR KO A549 cells were generated using the lentiCRISPR/CAS9-v2 system[80]. For both PARP14 and IFNAR1 pLentiCRISPR/CAS9-v2 constructs, a pair of oligos were phosphorylated, annealed, and inserted into pLentiCRISPRv2 (Addgene, plasmid 52961) between BsmBI restriction sites as described[80]. The sequences of the oligos are listed in S2 Table. The packaging of lentiviruses and transduction were performed as described previously [81]. In summary, pooled PARP14 KO NHDF cells or PARP14 KO or IFNAR KO A549 cell lines were generated by transduction with lentiviruses with lentiCRISPR/CAS9-v2-gRNAs. 72 hours post transduction, NHDF and A549 cells were treated with puromycin (2μg/ml) for 3 days. A549 cells were diluted to obtain single cell clones, while NHDF cells were pooled. PARP14 KO clones were screened by immunoblotting for PARP14. For IFNAR KO cells, ablation was confirmed by assessing infection sensitivity to IFNα. NHDF and A549 cells transduced with irrelevant gRNA (gRNA-NC) were used as negative controls.

### Conditioned media activity assay

WT or PARP14 KO A549 or NHDF cells were seeded in 12-well plates (10^5^ cells/well). 16 hours later, cells were mock transfected (wild-type cells) or transfected with 2 μl Lipofectamine 2000 reagent (Invitrogen) and 500 ng poly(I:C) (HMW, In*vivo*Gen) in 200 μl of DMEM in duplicate as per manufacturer’s protocol (for recipient WT and IFNAR KO A549 cells). To reduce the effect of the remaining poly(I:C) in the conditioned media, cell media were replaced with fresh media 2 h post transfection. At 6 h post transfection, cell media was collected and centrifuged to remove cell debris, and supernatants were collected as conditioned media. Recipient WT or IFNAR KO A549 cells were then incubated with one of the duplicate samples of conditioned media for 2 h prior to infection with eGFP-VSV (rM51R-M-EGFP) at an MOI of 1 PFU/cell. VSV titers in the supernatants were determined by counting plaques on Vero cells.

### IFN activity assay

WT or IFNAR KO A549 cells were incubated with 1 ml of IFNα-containing media (0, 1, 10, 100, 1000 units/ml) for 4 h prior to infection with eGFP-VSV (rM51R-M-EGFP) at an MOI of 1 PFU/cell. At 16 hpi, infected cells were monitored under an AMG-EVOS FL Digital Inverted Fluorescence Microscope (software version 15913). GFP fluorescence microscopy was performed with a light cube of GFP (Ex 470 nm/Em 525 nm) under a 4X objective lens (the scale bar on the image represents 1000 μm), and images of six different fields were taken for each condition. The quantification of GFP-VSV-infected cells was performed using ImageJ software (NIH) to count GFP (green) points. The means ± SEM of percentages of infected cells from six different fields (three each from two different replicate wells of a 12-well-plate) are shown.

### Real-time quantitative PCR (RT-qPCR) analysis

RNA was isolated from cells using Trizol (Thermo Fisher Scientific) via phase separation or Direct-Zol column purification (Zymo Research) as per manufacturer’s instructions. cDNA was prepared using MMLV-reverse transcriptase as per manufacturer’s instructions (Thermo Fisher Scientific). Quantitative PCR (qPCR) was performed on a QuantStudio3 real-time PCR system using PowerUp SYBR Green Master Mix (Thermo Fisher Scientific). qPCR primers are listed in S3 Table. Primers were designed to span an exon-exon junction when possible to prevent quantification of any residual genomic DNA. All qPCR reactions were run with a -RT control to confirm the lack of significant DNA contamination. Cycle thresholds were normalized to that of housekeeping gene hypoxanthine-guanine phosphoribosyltransferase (HPRT) by the following equation: Δ*C*_*T* =_ *C*_*T* (gene of interest)_—*C*_*T* (HPRT)_. All results are shown as a ratio to HPRT calculated as -2^Δ*CT*^.

### Immunoblotting

Total cells were lysed in sample buffer containing SDS, β-mercaptoethanol, protease/phosphatase inhibitor cocktails (Roche), PMSF, and universal nuclease (Thermo Fisher Scientific). Proteins were resolved on an SDS polyacrylamide gel, transferred to a polyvinylidene difluoride (PVDF) membrane, hybridized with a primary antibody, reacted with an infrared (IR) dye-conjugated secondary antibody, visualized using a LI-COR Odyssey Imager, and analyzed using Image Studio software (LI-COR). Primary antibodies used for immunoblotting include anti-FLAG monoclonal antibody (M2, 1:500, Millipore-Sigma); anti-PARP14 polyclonal antibodies (C-1, 1:500, Santa Cruz Biotechnology; HPA012063, 1:1000, Millipore-Sigma); anti-PAR monoclonal antibody (10H, 1:500, Trevigen); rabbit anti-MHV polyclonal antibody (1:10,000) [82]; and anti-actin monoclonal antibody (AC15, 1:10,000, Abcam). Secondary IR antibodies were purchased from LI-COR.

### Measurement of IFN protein levels

Supernatants from infected cells were collected at 12 hpi, and protein levels of IFNα and IFNβ were determined using the Luminex Protein Assay (Thermo Fisher Scientific) according to the manufacturer’s instructions.

### Fluorescence microscopy

BMDMs plated on glass cover slips were infected with GFP-expressing MHV. At 14 hpi, cells were fixed with 4% paraformaldehyde, and coverslips were transferred to a glass slide. Vectashield Antifade Mounting Media with DAPI (Vector Laboratories) was applied, and a second coverslip was overlaid. Slides were visualized on an Olympus IX-81 inverted fluorescence microscope (Olympus), and images were analyzed using SlideBook software (Meyers Instruments).

### Statistics

An unpaired two-tailed Student’s t-test was used to assess differences in mean values between groups, and graphs are expressed as mean ± SEM. MHV titers are presented as geometric mean ± SEM. The n value represents the number of biologic replicates for each figure. The n for WT and N1347A virus-infected samples were the same unless otherwise indicated. Significant p values are denoted with *p≤0.05, ** p≤0.01, *** p≤0.001.

## Supporting information

S1 Fig Virus replication and PARP expression in brain CD11b+ cells infected by WT and N1347A MHV. (A) Mice were infected as described in Methods with WT and N1347A MHV. Brain tissues were collected at 4 dpi, and CD11b+ cells were purified as described in Methods. Flow cytometry was used to assess purification efficacy. The data in (A) are from one representative experiment of three independent experiments; n = 4. (B,C) CD11b+ cells were purified, and RNA was isolated and analyzed for viral genomic RNA (gRNA) at indicated time points (B) or for PARP mRNA at 4 dpi (C). The data in (B,C) show one representative experiment of two independent experiments; n = 4 for WT and N1347A except N1347A at day 5 where n = 3. For naïve samples in (C), n = 2. Numbers above bars represent fold difference between WT and N1347A.
(TIF)

S2 Fig N1347A MHV virulence is restored in IFNAR^-/-^ but not MAVS^-/-^ mice. WT, MAVS^-/-^, or IFNAR^-/-^ C57BL/6 mice were infected as described in Methods and monitored for weight loss and survival over a 12-day period. WT, n = 5; MAVS^-/-^, n = 9 for WT and n = 11 for N1347A; IFNAR^-/-^, n = 3 for WT and n = 7 for N1347A.
(TIF)

S3 Fig The effect of PARP inhibitors on cellular metabolism and PARylation. (A,B) BMDMs were incubated with PARP inhibitors 3-AB (5 mM), XAV-939 (10 μM), or vehicle (0.25% DMSO) (A) or with PARP14-specific inhibitor compound 8K (5 μM) or vehicle (B). At 24 hours, cell viability was measured using an MTT assay as described in Methods. The data in (A,B) show one experiment representative of two independent experiments; n = 4. (C) DBT cells were treated with or vehicle (0.25% DMSO), 3-AB (5 mM), XAV-939 (10 μM), or 8K (5 μM). After 18 h, cell lysates were collected and immunoblotted for poly(ADP-ribose) (PAR) or for actin. The data in (C) show one experiment representative of at least two independent experiments.
(TIF)

S4 Fig siRNA knockdown of PARP mRNA in BMDMs. BMDMs were transfected with control siRNA (siCtl) or PARP-specific siRNA as described in Methods. Approximately 28 hours later, cells were infected with WT or N1347A MHV and collected at 18–22 hpi. RNA levels were determined by RT-qPCR with primers specific for each transcript and normalized to HPRT. The level of PARP mRNA in siRNA-treated cells was then normalized to expression in control siRNA-treated cells. The data show the combined results of two to five experiments; n = 9 for siPARP7, n = 6 for siPARP9, n = 6 for siPARP10, n = 12 for siPARP11, n = 15 for siPARP12.2, n = 9 for siPARP12.3, n = 12 for siPARP14.1, n = 12 for siPARP14.2.
(TIF)

S5 Fig PARP14 protein expression in PARP14^-/-^ and PARP14^+/-^ cells. BMDMs were infected with WT or N1347A MHV and collected at 12 hpi. Lysates were analyzed by immunoblotting with the indicated antibodies using a LI-COR Odyssey Imager. The data show the results of one experiment representative of two independent experiments.
(TIF)

S6 Fig PARP12 and PARP14 overexpression in DBT cells. (A) DBT cells were transfected with indicated plasmids and collected 24 hours after transfection. Lysates were analyzed by immunoblotting with the indicated antibodies using a LI-COR Odyssey Imager. The data are the results of one experiment representative of two independent experiments. FLAG/PARP12 was utilized as a positive control for the anti-FLAG blot. (B) DBT cells were transfected with plasmid expressing GFP, PARP14, or a PARP14 catalytic mutant (CM). Cells were collected at 24 hours after transfection, and PARP14 mRNA levels were determined by RT-qPCR and normalized to HPRT. The data in (B) show one experiment representative of two independent experiments; n = 3.
(TIF)

S7 Fig PARP14 is required for IFN-I production in NHDFs. (A) A pool of CRISPR/Cas9-gRNA-mediated PARP14 KO NHDF cells or of gRNA-NC-transduced control NHDF cells were collected and analyzed by immunoblotting for PARP14 protein. (B,C) A549 cells were treated with conditioned media for 2 h, VSV replication was analyzed by fluorescence microscopy at 16 hpi (B), and titers were determined by plaque assay on Vero cells (C). Titers were normalized to those in mock-treated wild-type cells. p-value markers in (C) represent comparisons of poly(I:C) (pI:C)-treated PARP14-KO cell conditioned media to poly(I:C)-treated WT cell conditioned media used to pretreat WT or IFNAR KO A549 cells. The data in (B) show a single experiment representative of three independent experiments, and the data in (C) show the combined results of three independent experiments; n = 3. (D) WT or IFNAR KO A549 cells were pre-treated for 4 hours with varying amounts of IFNα and infected with eGFP-VSV (rM51R-M-EGFP) at an MOI of 1 PFU/cell. Quantification of eGFP was performed directly using fluorescence microscopy and ImageJ software. Shown is virus titer in IFN-treated cells relative to titer in mock-treated cells as determined by percent eGFP-positive cells. The data in (D) show one experiment representative of two independent experiments; n = 6.
(TIFF)

S1 Table siRNA sequences. Sequences of small interfering RNAs used to knockdown gene expression are listed.
(TIFF)

S2 Table gRNA sequences. Guide RNA sequences used to develop lentiCRISPR/CAS9-v2-mediated knockout pools and clones of cells are listed.
(TIFF)

S3 Table Quantitative real-time qPCR sequences. Primer sequences used to quantify transcription of specific genes are listed.
(TIFF)
