# Supplementary figures and images for: The coronavirus macrodomain is required to prevent PARP-mediated inhibition of virus replication and enhancement of IFN expression
Source: PLoS Pathog. 2019 May 16;15(5):e1007756. doi: 10.1371/journal.ppat.1007756 (PMC6521996; doi:10.1371/journal.ppat.1007756)

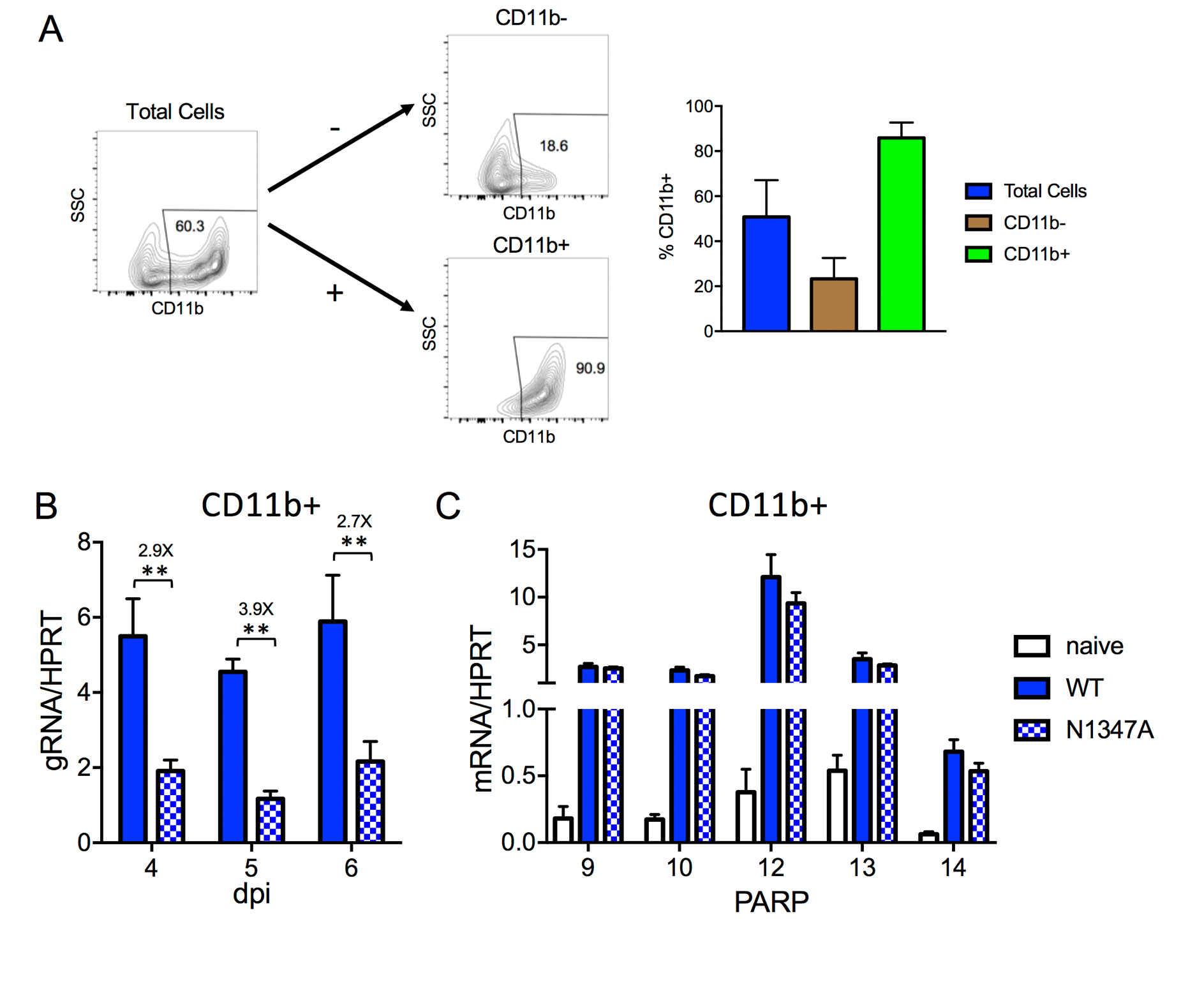

Supplement: S1 Fig — (A) Mice were infected as described in Methods with WT and N1347A MHV. Brain tissues were collected at 4 dpi, and CD11b+ cells were purified as described in Methods. Flow cytometry was used to assess purification efficacy. The data in (A) are from one representative experiment of three independent experiments; n = 4. (B,C) CD11b+ cells were purified, and RNA was isolated and analyzed for viral genomic RNA (gRNA) at indicated time points (B) or for PARP mRNA at 4 dpi (C). The data in (B,C) show one representative experiment of two independent experiments; n = 4 for WT and N1347A except N1347A at day 5 where n = 3. For naïve samples in (C), n = 2. Numbers above bars represent fold difference between WT and N1347A. (TIF) [file ppat.1007756.s001.tif]

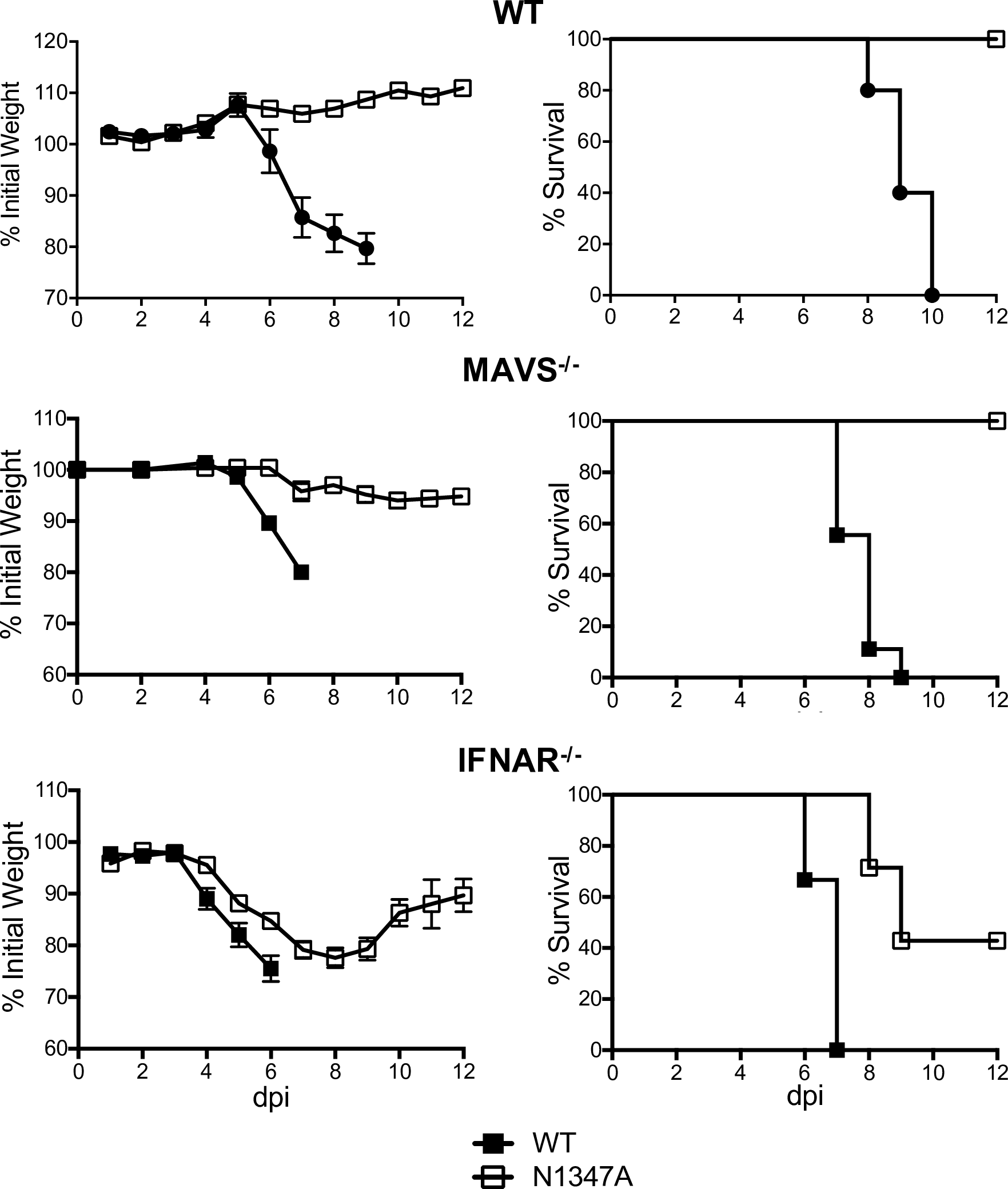

Supplement: S2 Fig — WT, MAVS-/-, or IFNAR-/- C57BL/6 mice were infected as described in Methods and monitored for weight loss and survival over a 12-day period. WT, n = 5; MAVS-/-, n = 9 for WT and n = 11 for N1347A; IFNAR-/-, n = 3 for WT and n = 7 for N1347A. (TIF) [file ppat.1007756.s002.tif]

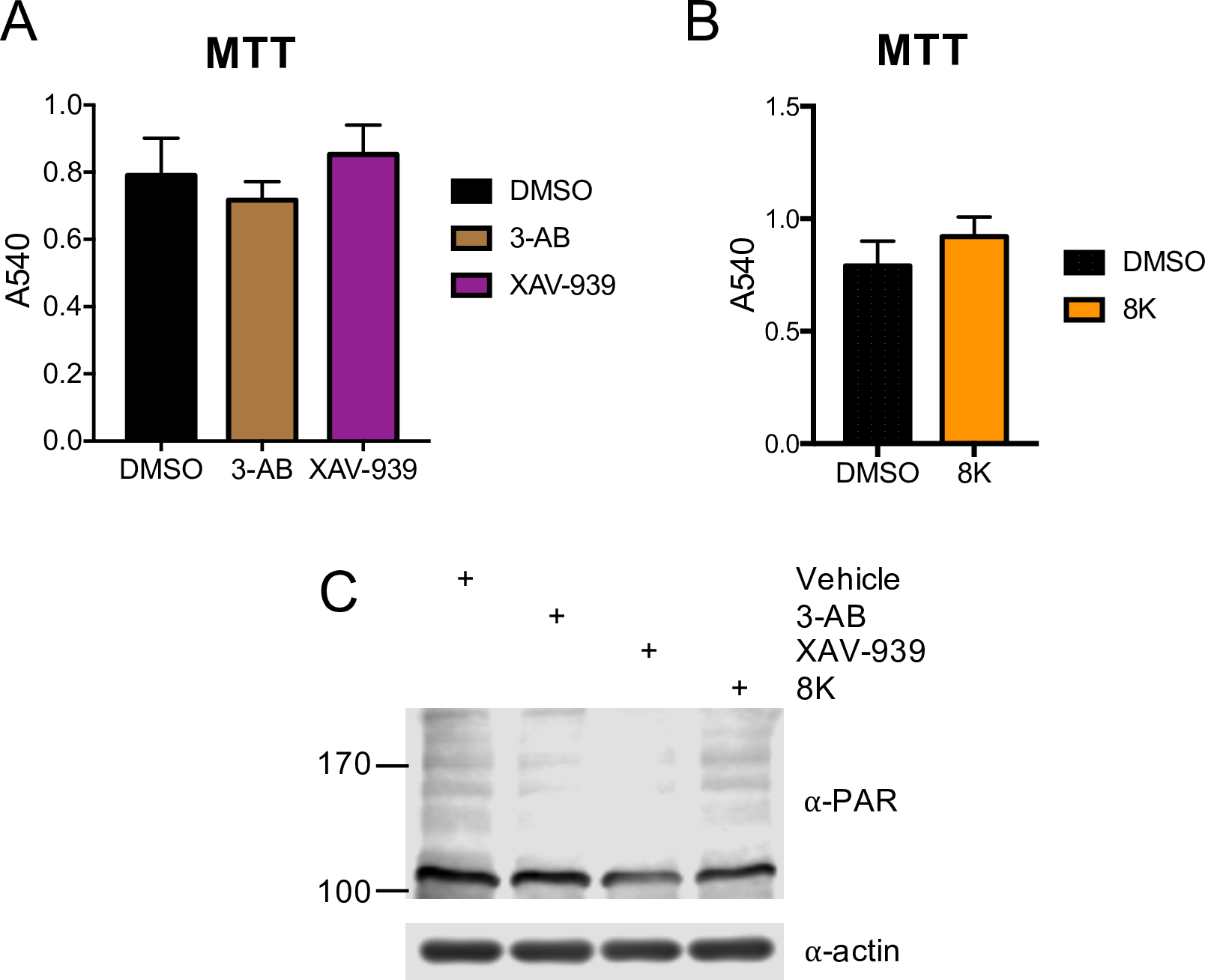

Supplement: S3 Fig — (A,B) BMDMs were incubated with PARP inhibitors 3-AB (5 mM), XAV-939 (10 μM), or vehicle (0.25% DMSO) (A) or with PARP14-specific inhibitor compound 8K (5 μM) or vehicle (B). At 24 hours, cell viability was measured using an MTT assay as described in Methods. The data in (A,B) show one experiment representative of two independent experiments; n = 4. (C) DBT cells were treated with or vehicle (0.25% DMSO), 3-AB (5 mM), XAV-939 (10 μM), or 8K (5 μM). After 18 h, cell lysates were collected and immunoblotted for poly(ADP-ribose) (PAR) or for actin. The data in (C) show one experiment representative of at least two independent experiments. (TIF) [file ppat.1007756.s003.tif]

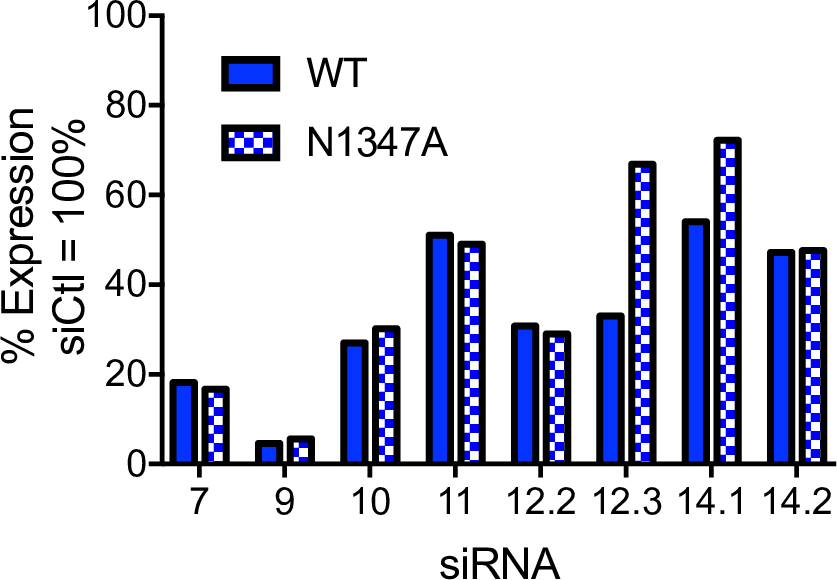

Supplement: S4 Fig — BMDMs were transfected with control siRNA (siCtl) or PARP-specific siRNA as described in Methods. Approximately 28 hours later, cells were infected with WT or N1347A MHV and collected at 18–22 hpi. RNA levels were determined by RT-qPCR with primers specific for each transcript and normalized to HPRT. The level of PARP mRNA in siRNA-treated cells was then normalized to expression in control siRNA-treated cells. The data show the combined results of two to five experiments; n = 9 for siPARP7, n = 6 for siPARP9, n = 6 for siPARP10, n = 12 for siPARP11, n = 15 for siPARP12.2, n = 9 for siPARP12.3, n = 12 for siPARP14.1, n = 12 for siPARP14.2. (TIF) [file ppat.1007756.s004.tif]

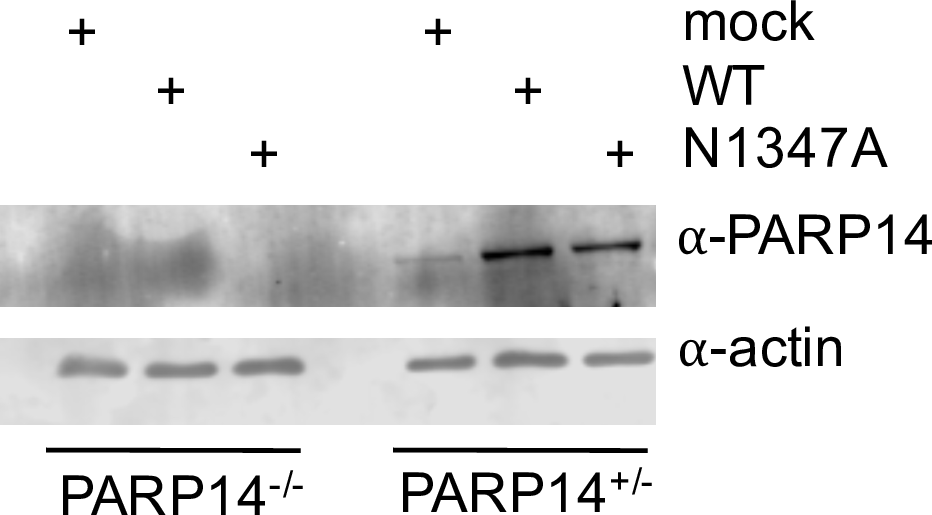

Supplement: S5 Fig — BMDMs were infected with WT or N1347A MHV and collected at 12 hpi. Lysates were analyzed by immunoblotting with the indicated antibodies using a LI-COR Odyssey Imager. The data show the results of one experiment representative of two independent experiments. (TIF) [file ppat.1007756.s005.tif]

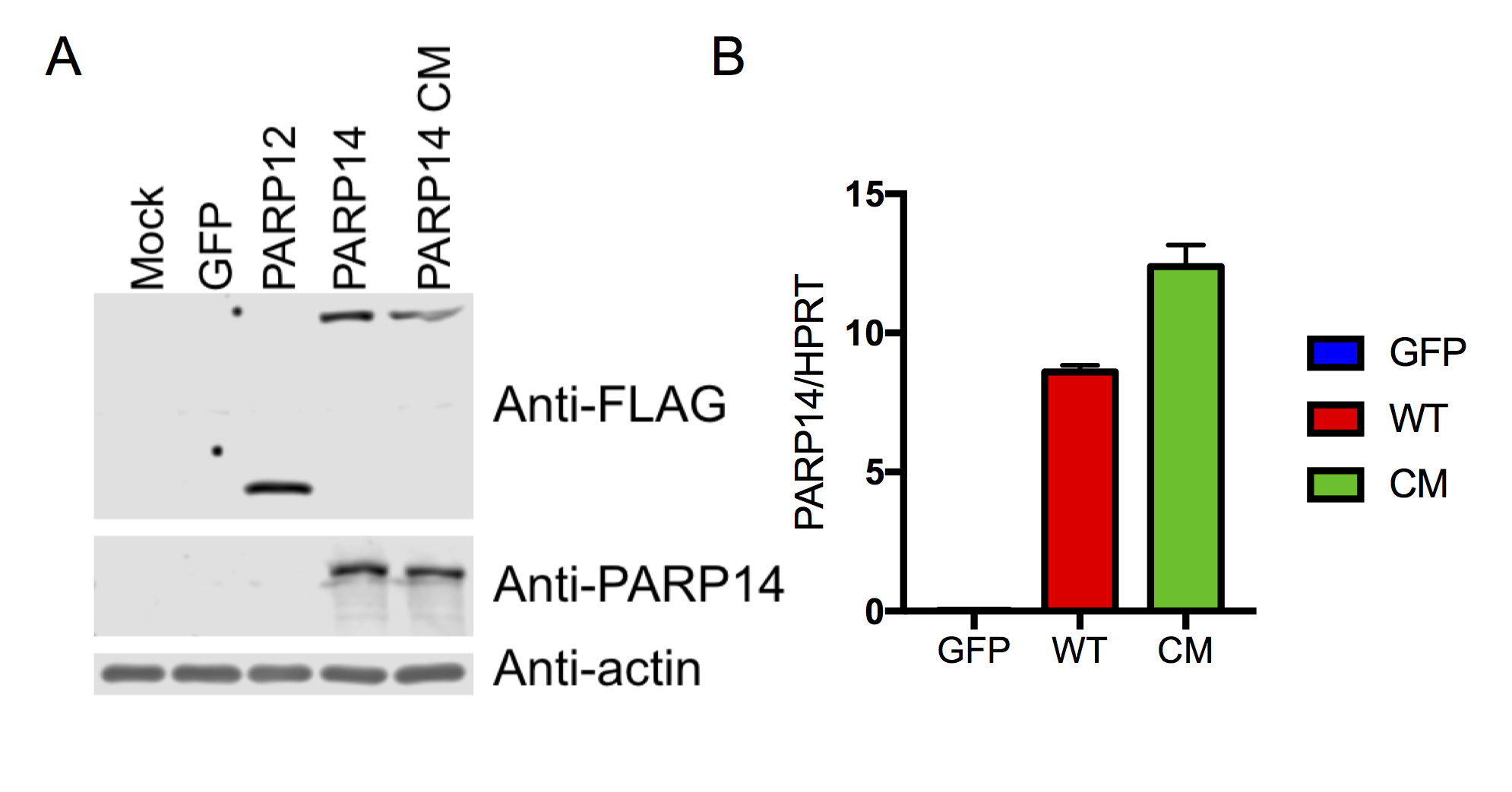

Supplement: S6 Fig — (A) DBT cells were transfected with indicated plasmids and collected 24 hours after transfection. Lysates were analyzed by immunoblotting with the indicated antibodies using a LI-COR Odyssey Imager. The data are the results of one experiment representative of two independent experiments. FLAG/PARP12 was utilized as a positive control for the anti-FLAG blot. (B) DBT cells were transfected with plasmid expressing GFP, PARP14, or a PARP14 catalytic mutant (CM). Cells were collected at 24 hours after transfection, and PARP14 mRNA levels were determined by RT-qPCR and normalized to HPRT. The data in (B) show one experiment representative of two independent experiments; n = 3. (TIF) [file ppat.1007756.s006.tif]

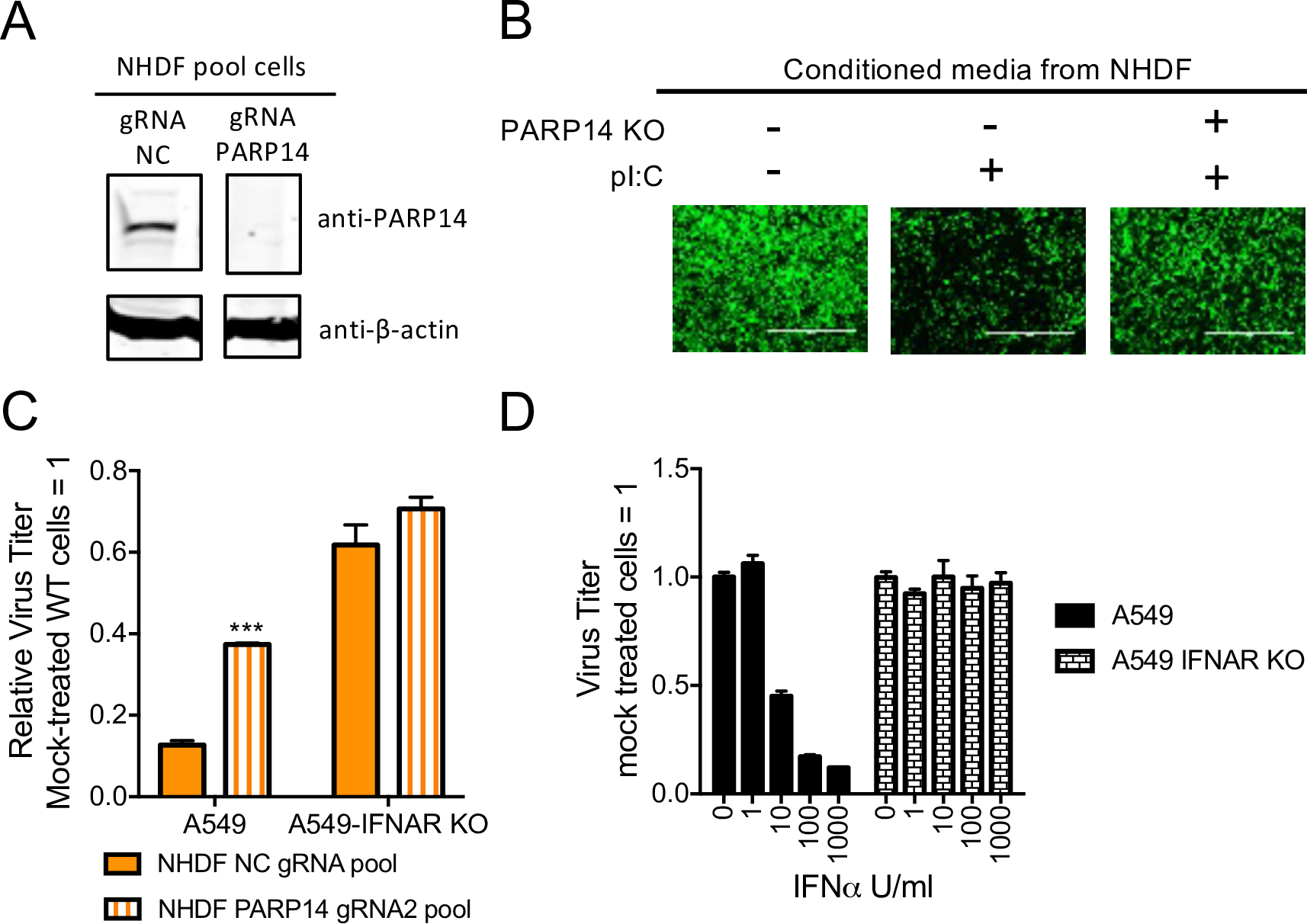

Supplement: S7 Fig — (A) A pool of CRISPR/Cas9-gRNA-mediated PARP14 KO NHDF cells or of gRNA-NC-transduced control NHDF cells were collected and analyzed by immunoblotting for PARP14 protein. (B,C) A549 cells were treated with conditioned media for 2 h, VSV replication was analyzed by fluorescence microscopy at 16 hpi (B), and titers were determined by plaque assay on Vero cells (C). Titers were normalized to those in mock-treated wild-type cells. p-value markers in (C) represent comparisons of poly(I:C) (pI:C)-treated PARP14-KO cell conditioned media to poly(I:C)-treated WT cell conditioned media used to pretreat WT or IFNAR KO A549 cells. The data in (B) show a single experiment representative of three independent experiments, and the data in (C) show the combined results of three independent experiments; n = 3. (D) WT or IFNAR KO A549 cells were pre-treated for 4 hours with varying amounts of IFNα and infected with eGFP-VSV (rM51R-M-EGFP) at an MOI of 1 PFU/cell. Quantification of eGFP was performed directly using fluorescence microscopy and ImageJ software. Shown is virus titer in IFN-treated cells relative to titer in mock-treated cells as determined by percent eGFP-positive cells. The data in (D) show one experiment representative of two independent experiments; n = 6. (TIFF) [file ppat.1007756.s007.tiff]

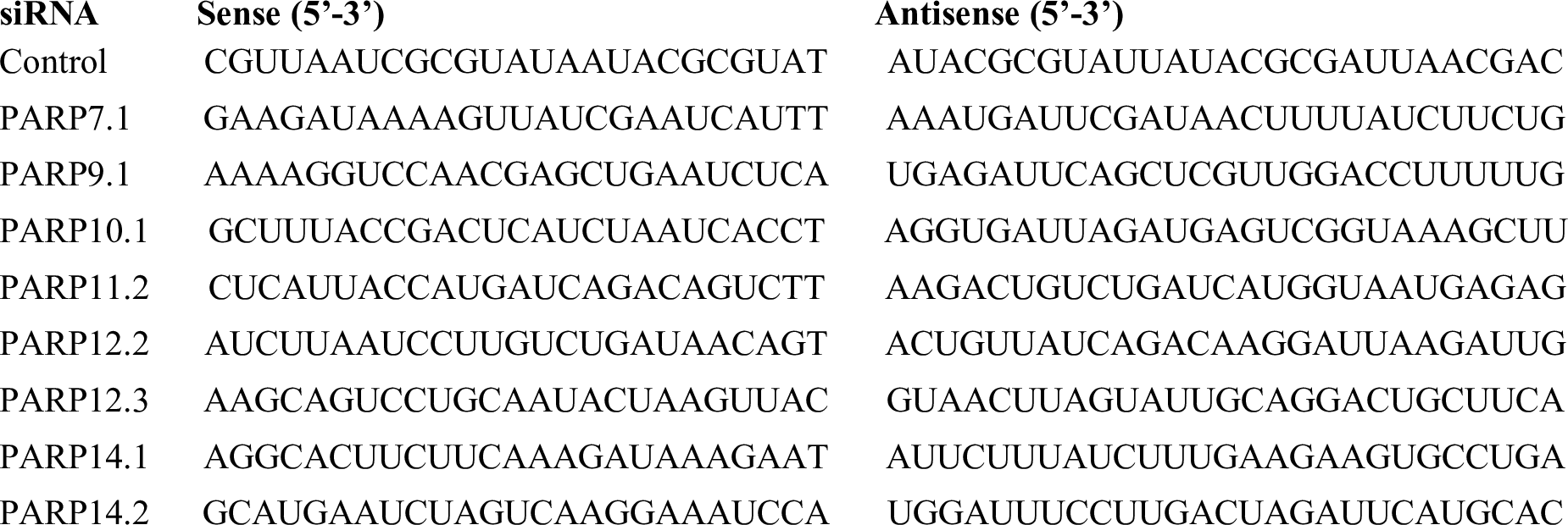

Supplement: S1 Table — Sequences of small interfering RNAs used to knockdown gene expression are listed. (TIFF) [file ppat.1007756.s008.tiff]

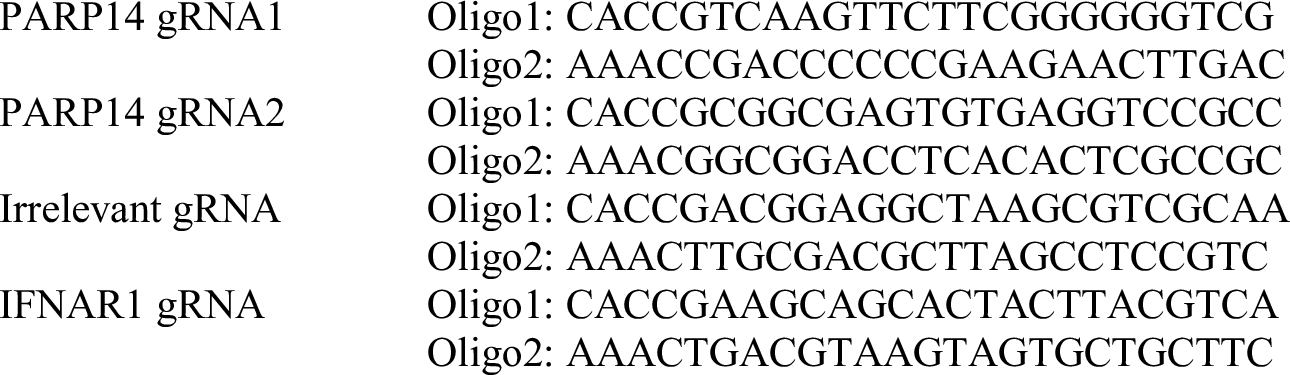

Supplement: S2 Table — Guide RNA sequences used to develop lentiCRISPR/CAS9-v2-mediated knockout pools and clones of cells are listed. (TIFF) [file ppat.1007756.s009.tiff]

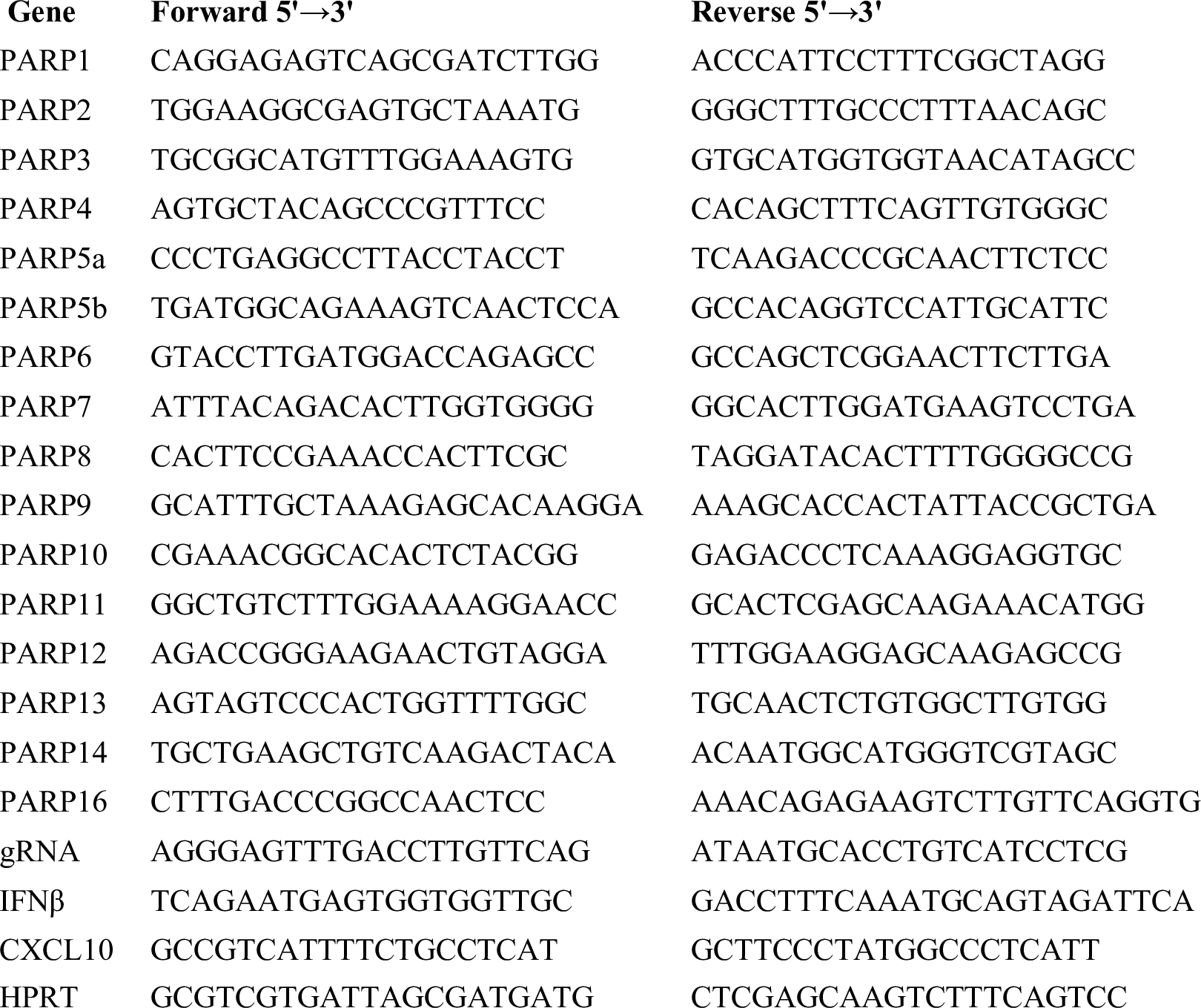

Supplement: S3 Table — Primer sequences used to quantify transcription of specific genes are listed. (TIFF) [file ppat.1007756.s010.tiff]
